# MEK Is a Potential Indirect Target in Subtypes of Head and Neck Cancers

**DOI:** 10.3390/ijms24032782

**Published:** 2023-02-01

**Authors:** Bianka Gurbi, Diána Brauswetter, Kinga Pénzes, Attila Varga, Tibor Krenács, Kornél Dános, Ede Birtalan, László Tamás, Miklós Csala

**Affiliations:** 1 Department of Molecular Biology, Semmelweis University, H-1094 Budapest, Hungary; 2 MTA-SE Pathobiochemistry Research Group, Semmelweis University, H-1094 Budapest, Hungary; 3 Department of Pathology and Experimental Cancer Research, Semmelweis University, H-1085 Budapest, Hungary; 4 Department of Oto-Rhino-Laryngology, Head and Neck Surgery, Semmelweis University, H-1083 Budapest, Hungary; 5 Department of Voice, Speech and Swallowing Therapy, Faculty of Health Sciences, Semmelweis University, H-1088 Budapest, Hungary

**Keywords:** head-and-neck cancer, epidermal growth factor receptor, MEK, feedback loop

## Abstract

The poor prognosis of head-and-neck squamous cell carcinoma (HNSCC) is partly due to the lack of reliable prognostic and predictive markers. The Ras/Raf/MEK/ERK signaling pathway is often activated by overexpressed epidermal growth factor receptor (EGFR) and stimulates the progression of HNSCCs. Our research was performed on three human papillomavirus (HPV)-negative HNSCC-cell lines: Detroit 562, FaDu and SCC25. Changes in cell viability upon EGFR and/or MEK inhibitors were measured by the MTT method. The protein-expression and phosphorylation profiles of the EGFR-initiated signaling pathways were assessed using Western-blot analysis. The EGFR expression and pY1068-EGFR levels were also studied in the patient-derived HNSCC samples. We found significant differences between the sensitivity of the tumor-cell lines used. The SCC25 line was found to be the most sensitive to the MEK inhibitors, possibly due to the lack of feedback Akt activation through EGFR. By contrast, this feedback activation had an important role in the FaDu cells. The observed insensitivity of the Detroit 562 cells to the MEK inhibitors might have been caused by their PIK3CA mutation. Among HNSCC cell lines, EGFR-initiated signaling pathways are particularly versatile. An ERK/EGFR feedback loop can lead to Akt-pathway activation upon MEK inhibition, and it is related not only to increased amounts of EGFR but also to the elevation of pY1068-EGFR levels. The presence of this mechanism may justify the combined application of EGFR and MEK inhibitors.

## 1. Introduction

According to Globocan 2020, head-and-neck squamous cell carcinomas (HNSCCs) are the sixth most common types of cancer worldwide, with nearly 900,000 new cases in 2020 [1]. The major causative agents in the development of HNSCC are alcohol consumption, exposure to environmental pollutants, the chewing of various areca-nut products, tobacco, and human papillomavirus (HPV) infection. The latter, as well as being a leading cause of cervical cancer, contributes to more than the 50% of HNSCC progression. The prognosis of HPV-positive tumors is better than that of HPV-negative tumors in general. Furthermore, their response for non-surgical treatments (chemotherapy, radiotherapy, radio-chemotherapy, epidermal growth factor receptor (EGFR) targeted therapy) is more favorable [2].

Despite the well characterized genomic alterations (The Cancer Genome Atlas, TCGA) and many clinical trials with targeted therapeutic agents, only the EGFR inhibitor cetuximab and the immune-checkpoint PD-1 (programmed cell death protein 1) inhibitors pembrolizumab and nivolumab are approved by the FDA as molecular targeted therapies in the treatment of HNSCCs. The monoclonal antibody cetuximab does not currently have a reliable predictive biomarker. Surgery, supplemented with (chemo)radiotherapy, or combined chemoradiotherapy +/− salvage surgery is still the first-line therapy in locoregionally advanced cancers. Molecular therapies are only used in the recurrent/metastatic setting or in platinum-resistant diseases [3,4].

It is clear that the receptor tyrosine kinase EGFR has an important role in the pathogenesis of HNSCCs as its overexpression was observed even in the early stages of tumorigenesis. Nevertheless, it seems that the expression of the EGFR protein is not appropriate for predicting the effectiveness of anti-EGFR agents. There is also controversy in the literature as to whether EGFR expression has prognostic value in HNSCCs; significant associations with both better and worse prognoses have been found in different trials. Several studies have propounded the level of Y1068-EGFR phosphorylation as a predictive or prognostic factor; however, its actual role has been equally disputed [5,6,7,8,9].

To inhibit a downstream target such as MEK, which plays an important role in many steps of tumorigenesis, could also be a significant opportunity. The MEK inhibitor trametinib is already used in the treatment of metastatic melanoma harboring the BRAF mutation and as a part of a drug combination in many locally advanced or metastatic solid tumors [10,11].

In 2016, Stockley et al. proved that patients participating in a genotype-matched study have a significantly better survival compared to those in classic histology/anatomy-based studies. This observation shed light on the importance of identifying the most dominant pathways and the driver genes when selecting the proper treatment [12]. In this pathway analysis, attention needs to be paid to the potential feedback mechanisms that can cause pathway shift or drug resistance. One of the most important and most frequently studied feedback mechanisms is the upregulation of the EGFR/PI3K/Akt pathway upon MEK inhibition through the CDC25 phosphatase protein [13]. The use of trametinib in combination with EGFR inhibitors can be a solution to prevent this upregulation and to evade drug resistance. However, appropriate biomarkers are needed for selecting the correct drug combination.

In this study, our aim was to investigate the potential role of Y1068EGFR phosphorylation as a prognostic and predictive biomarker in HNSCCs using both formalin-fixed paraffin-embedded (FFPE) tissue samples and tumor-derived cell lines. In addition, by examining the most commonly studied proteins of the EGFR pathway, we established a theoretical pathway model describing the supposed resistance mechanisms to EGFR and MEK inhibitors in the HNSCC cell lines used.

## 2. Results

### 2.1. EGFR and pY1068-EGFR Protein Levels in Patient-Derived HNSCC Samples

**Figure 1:**
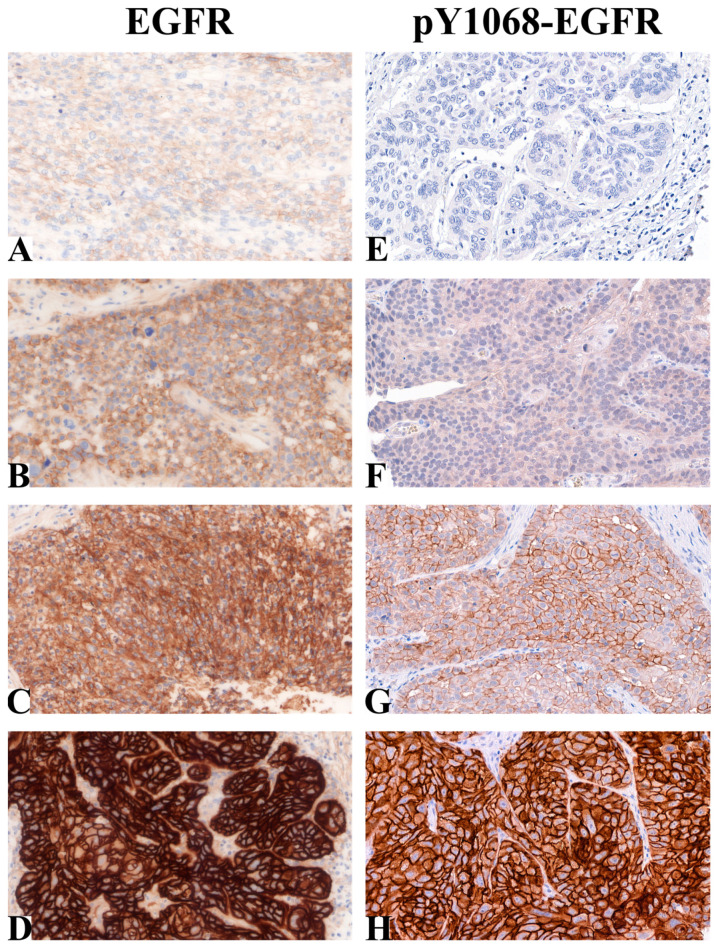
Immunohistochemical analysis of EGFR and pY1068-EGFR levels. (**A**) Negative, (**B**) low, (**C**) intermediate and (**D**) high EGFR levels in HNSCC tissue samples. (**E**) Negative, (**F**) low, (**G**) intermediate and (**H**) high pY1068-EGFR levels in HNSCC tissue samples (magnification: 40×).

Following immunohistochemical staining, 84 of the 97 patient samples were evaluable for EGFR and 63 for pY1068-EGFR. Typical tissue samples with low protein levels of EGFR (A,B), high EGFR (C,D), low pY1068-EGFR (E–G) and high pY1068-EGFR (H) are shown in Figure 1.

A high EGFR expression (EGFR^H^) was found in 78/84 (92.3%) of the HNSCC samples. The EGFR status did not correlate with disease-specific survival (DSS, *p* = 0.551), tumor localization (*p* = 0.369), tumor size (*p* = 0.690), lymph-node metastasis (*p* = 0.525), distant metastasis (*p* = 0.522), stage (*p* = 0.182), smoking (*p* = 0.866) or alcohol consumption (*p* = 0.707). However, we found a significant association with tumor grade (*p* = 0.031); in other words, high EGFR expression was associated with higher grade.

**Figure 2:**
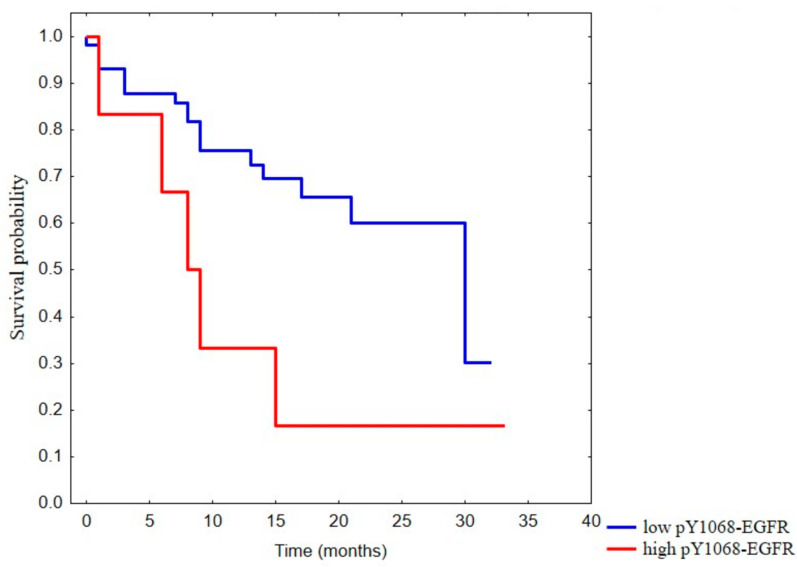
Disease-specific survival (DSS) according to pY1068-EGFR positivity. Kaplan–Meier plots of overall survival according to the groups of low (blue) or high (red) pY1068-EGFR levels (*p* = 0.036).

Of the sixty-three tumor samples, six (9.5%) showed high pY1068-EGFR levels (pY1068-EGFR^H^). Furthermore, the pY1068-EGFR status was correlated with the DSS (*p* = 0.036); that is, increases in pY1068-EGFR-expression levels were associated with poorer prognoses (Figure 2). However, the pY1068-EGFR status did not correlate with tumor localization (*p* = 0.748), tumor size (*p* = 0.303), distant metastasis (*p* = 0.406), stage (*p* = 0.273), grade (*p* = 0.745), smoking (*p* = 0.182) or alcohol consumption (*p* = 0.951). Nevertheless, we found a statistically significant association with lymph-node metastasis (*p* = 0.024), although this difference has no biological relevance due to the low case numbers.

### 2.2. The Effects of EGFR Inhibitors on Head-and-Neck-Cancer-Cell Viability

To create an in vitro cellular model of head-and-neck cancers, we selected three head-and-neck-cancer-cell lines from different locations (Detroit 562: pharynx, FaDu: hypopharynx and SCC25: tongue) harboring different genetic mutations (Detroit 562: *TP53*, *CDKN2A* and *PIK3CA* mutations, FaDu: *TP53*, *CDKN2A* and *SMAD4* mutations and SCC25: *TP53* and *CDKN2A* mutations) [14]. The HNSCC cell viability was analyzed by performing a MTT assay after 72 h of erlotinib and afatinib treatment at different concentrations. The selected EGFR tyrosine kinase inhibitors (TKIs) were approved for the treatment of several tumor types [15,16].

**Figure 3:**
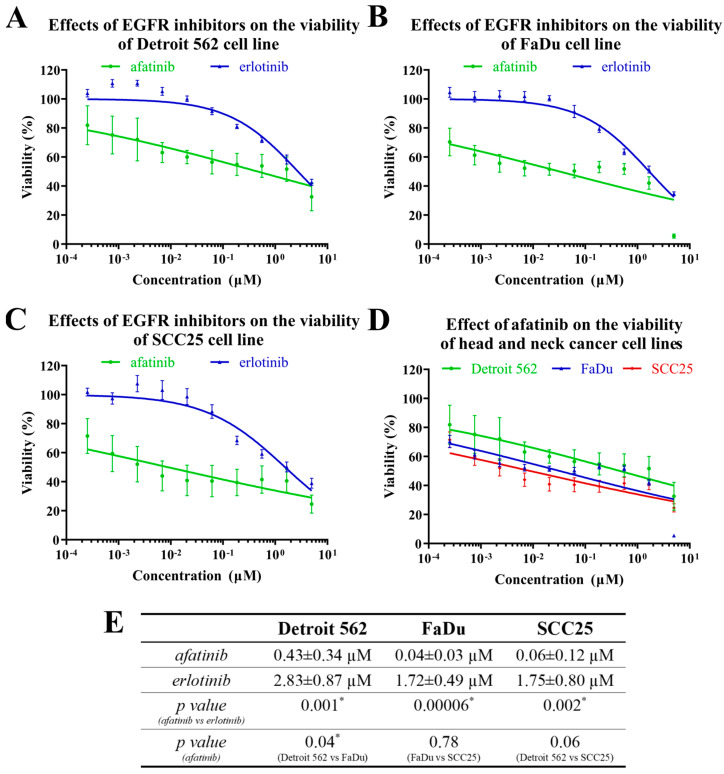
The effects of EGFR inhibitors on head-and-neck-cancer-cell viability. Cell lines were treated with afatinib or erlotinib at different concentrations for 72 h and cell viability was determined by MTT assay. The IC_50_ curves of the effects of the inhibitors on Detroit 562 (**A**), FaDu (**B**) and SCC25 (**C**) cell lines are shown. The IC_50_ curves of the effects of the afatinib on the three HNSCC cell lines are shown in a common diagram (**D**) for better comparison. (**E**) The IC_50_ values for the effects of the EGFR inhibitors on Detroit 562, FaDu and SCC25 cells. All data are represented as the mean ± SD of five independent measurements. The IC_50_ values (**E**) of the two inhibitors were compared to each other and Student’s *t*-tests were performed for statistically significant differences; * *p* < 0.05 afatinib vs. erlotinib and one afatinib-treated cell line vs. another.

Our results showed that both the EGFR inhibitors reduced the cell viability in a dose-dependent manner; however, the afatinib was a more potent inhibitor of tumor-cell viability than the erlotinib. The afatinib reduced the cell viability at the lowest investigated concentration (0.25 nM), while the erlotinib exerted its cytotoxic effect only over 62 nM in all the cell lines. Neither the erlotinib nor the afatinib caused complete cell death, even at the highest concentration, of 5 µM, in the Detroit 562 and SCC25 cells. However, the afatinib almost abolished the viability of the FaDu cells at the highest concentration applied (Figure 3A–C). We also observed a significant difference between the cancer-cell lines regarding the effects of the two EGFR inhibitors (Figure 3E). The FaDu and SCC25 cells were more sensitive than the Detroit 562 to both EGFR inhibitors, suggesting that the former cell lines are far more dependent on EGFR signaling than Detroit 562 cells (Figure 3D,E).

A comparison of the two EGFR inhibitors revealed that afatinib reduces HNSCC-cell viability more efficiently than erlotinib. A first-generation EGFR TKI, erlotinib is a competitive antagonist that binds reversibly to the ATP-binding site of the tyrosine kinase domain of EGFR. By contrast, the second-generation EGFR TKI, afatinib, inhibits both EGFR and human epidermal growth factor receptor 2 (HER2) irreversibly. In addition, afatinib inhibits several members of the EGFR family, which may explain its stronger effect on the HNSCC-cell viability [16,17,18].

### 2.3. The Effects of MEK Inhibitors on the Viability of Head-and-Neck-Cancer-Cell Lines

Similarly to the EGFR inhibitors, the two selected MEK inhibitors (selumetinib and trametinib), which are included in a clinical-phase study on several tumor types [19], were also tested for their effects on cell viability.

**Figure 4:**
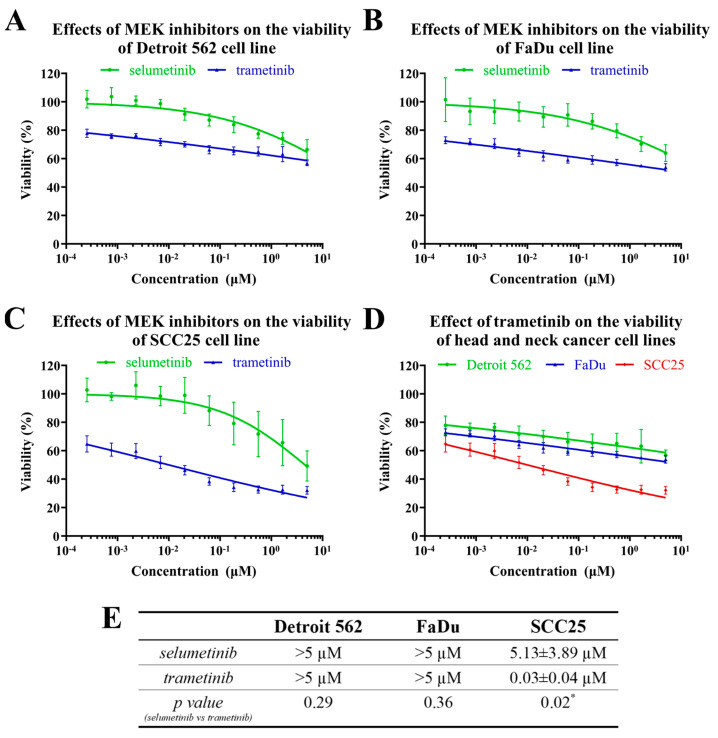
The effects of MEK inhibitors on head-and-neck-cancer-cell viability. The HNSCC cell lines were treated with selumetinib or trametinib at different concentrations for 72 h and cell viability was determined by MTT assay. The IC_50_ curves of the effects of the inhibitors on Detroit 562 (**A**), FaDu (**B**) and SCC25 (**C**) cell lines are shown. The IC_50_ curves of the effects of the trametinib on the three HNSCC cell lines are shown in a common diagram (**D**) for better comparison. (**E**) The IC_50_ values of the effects of the MEK inhibitors on Detroit 562, FaDu and SCC25 cells. All data are represented as the mean ± SD of five independent measurements. The IC_50_ values (**E**) of the two inhibitors were compared to each other and Student’s *t*-tests were performed for statistically significant differences; * *p* < 0.05 selumetinib vs. trametinib.

The treatments with selumetinib or trametinib only moderately decreased the viability of the Detroit 562 and FaDu cells; however, the SCC25 cells were fairly sensitive to both MEK inhibitors (Figure 4A–E). The trametinib was more effective than the selumetinib, which only decreased the cell viability at higher doses (IC_50_ values in SCC25 cells: 30 nM and 5.13 µM, respectively; *p* = 0.02; Figure 4E). Although the SCC25 cells were sensitive to the MEK-inhibitor treatments, neither the selumetinib nor the trametinib could reach complete cell death, as the maximum response values were 50% and 70%, respectively (Figure 4C).

These results clearly show that the SCC25 cell line was the most sensitive to the MEK-inhibitor treatment, and the trametinib proved to be a more potent inhibitor of cell proliferation than the selumetinib in these cellular models. While selumetinib is an effective, highly selective MEK1 inhibitor, trametinib has been shown to be active at sub-nanomolar concentrations against both purified MEK1 and MEK2 kinases, which may underlie its stronger effect on HNSCC-cell viability [19].

### 2.4. The Effects of EGFR- and MEK-Inhibitor Combinations on the Viability of Head-and-Neck-Cancer-Cell Lines

As the most effective EGFR and MEK inhibitors in our experiments, afatinib and trametinib were selected for further investigations using combination treatments. The viability of the HNSCC cells was assessed in parallel experiments by using a MTT assay after 72 h of treatment with afatinib + trametinib in a 1:1 ratio at different concentrations.

**Figure 5:**
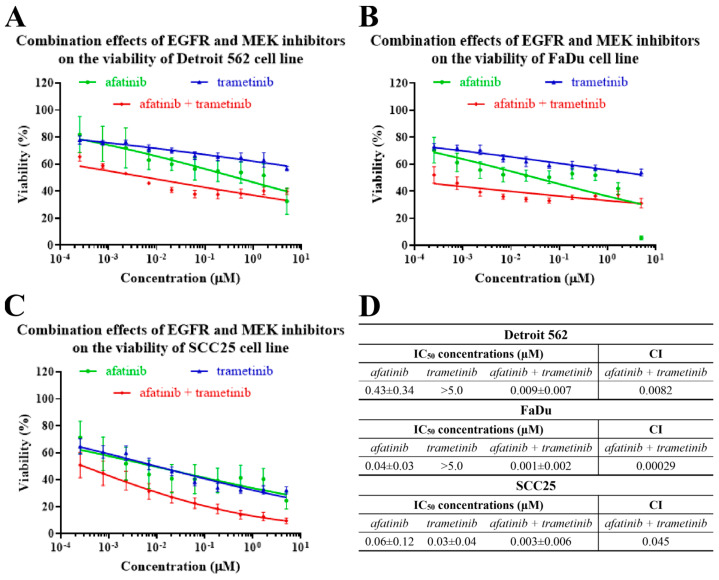
The effects of EGFR-inhibitor (afatinib) and MEK-inhibitor (trametinib) combinations on cell viability. The HNSCC cell lines were analyzed in parallel by MTT after 72 h of treatment with afatinib + trametinib (1:1) at different concentrations. The IC_50_ curves of the effects of the inhibitors on Detroit 562 (**A**), FaDu (**B**) and SCC25 (**C**) cell lines. (**D**) The IC_50_ values and combination indexes (CIs) of the effects of the different drug combinations on Detroit 562, FaDu and SCC25 cells. All data are represented as the mean ± SD of five independent measurements. The CIs were generated by CompuSyn, CI < 0.75 indicates synergism; CI between 0.75 and 1.25 indicates additive effects, and CI > 1.25 indicates antagonism.

Strong synergistic effects were observed in all the studied cell lines with the combination treatments (Figure 5). In the Detroit 562 cell line, the inhibitory effects of the afatinib and the trametinib in combination were stronger than the impact on the cell viability of the trametinib alone. However, notable differences between the combination treatment and the afatinib alone were observed only in a narrow concentration range. The Detroit 562 cells showed resistance to afatinib, and even the combination with trametinib did not decrease the IC_50_ values to an appropriate level (Figure 5A,D). By contrast, in the FaDu cells, the co-administration of afatinib and trametinib showed a stronger inhibitory effect on the cell viability at all the concentrations when compared to the trametinib alone, and at concentrations below 1.67 µM when compared to the afatinib alone. Moreover, a synergism was observed between the two substances at low concentrations, suggesting that a significant dose reduction could be achieved with combination therapy (Figure 5B,D). In the case of the SCC25, the combination of afatinib with trametinib had a stronger inhibitory effect on the viability than the two agents administered alone at concentrations above 0.19 µM. Based on this observation, we can conclude that the SCC25 cell line is highly sensitive to monotherapy, and unnecessary combination treatments would only increase drug toxicity (Figure 5C,D).

### 2.5. Protein Expression and Phosphorylation Analysis of Head and Neck Cancer Cell Lines

**Figure 6:**
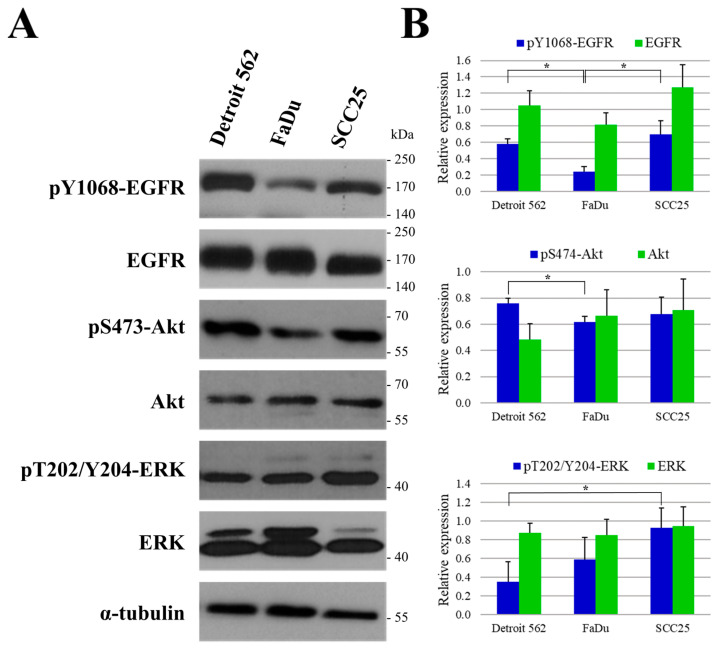
Protein expression and phosphorylation in head-and-neck squamous cell carcinoma (HNSCC) cell lines. (**A**) Cells were subjected to Western blot analysis with antibodies against pY1068-EGFR, EGFR, pS473-Akt, Akt, pT202/Y204-ERK, ERK and the loading control, α-tubulin. (**B**) Densitometry analysis of pY1068-EGFR, EGFR, pS473-Akt, Akt, pT202/Y204-ERK and ERK expression in Detroit 562, FaDu and SCC25 cells. The expressions of all proteins were normalized to expression of α-tubulin. All data are presented as mean ± SD of three independent experiments. The pY1068-EGFR, EGFR, pS473-Akt, Akt, pT202/Y204-ERK and ERK levels of the cell lines were compared to each other, and Student’s *t*-tests were performed for statistically significant differences; * *p* < 0.05 one cell line vs. another.

Next, using our selected HNSCC cell lines, we analyzed the expression and phosphorylation levels of EGFR, and two prominent EGFR signaling related kinases, namely Akt and ERK proteins (Figure 6). Y1068 autophosphorylation site of EGFR was monitored as it is known to activate downstream signaling cascades, such as the MEK/ERK and PI3K/Akt pathways most frequently [20]. For the phosphorylation sites of ERK (T202/Y204) and Akt (S473) to be assessed, those involved in the activation of these pathways were chosen [20,21].

We did not find any significant difference between the three cell lines regarding the expression levels of the investigated proteins (*p* > 0.05). However, protein phosphorylation was not uniform, as the amount of pY1068-EGFR was significantly lower in FaDu cells than in the other two cell lines (Detroit 562 *p* = 0.002, SCC25 *p* = 0.011). Furthermore, the level of pS473-Akt was significantly higher in Detroit 562 than in FaDu cells (*p* = 0.013), and the level of pT202/Y204-ERK was significantly elevated in SCC25 compared to Detroit 562 (*p* = 0.028). It is concluded that PI3K/Akt signaling pathway is more active in Detroit 562 cells, while in SCC25 cells, the activity of MEK/ERK signaling pathway is the strongest. The activity of these two signaling pathways appears to be nearly identical in FaDu cells.

In summary, while there is no statistically significant difference between the expression of EGFR, Akt and ERK in the investigated cell lines, the pattern of phosphorylation/activity levels of these proteins differ significantly, suggesting diverse signaling network backgrounds in the three cell lines.

### 2.6. The Responses of Head-and-Neck-Cancer-Cell Lines to Trametinib Treatment

**Figure 7:**
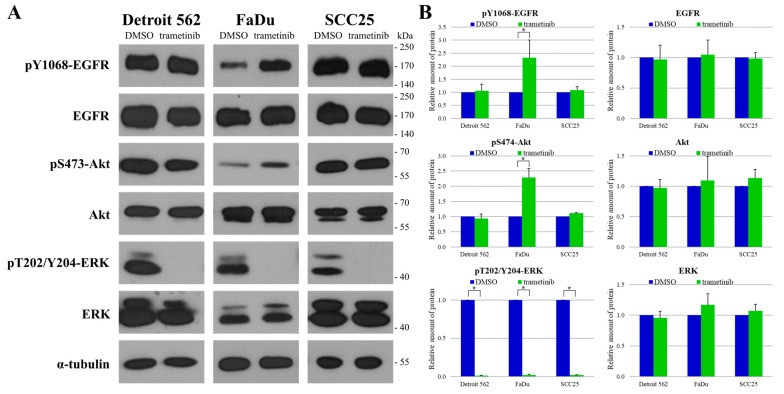
Changes in protein expression and phosphorylation after trametinib treatment in HNSCC cell lines. (**A**) Treated cells were subjected to Western blot analysis with antibodies against pY1068-EGFR, EGFR, pS473-Akt, Akt, pT202/Y204-ERK, ERK and the loading control, α-tubulin. (**B**) Densitometry analysis of pY1068-EGFR, EGFR, pS473-Akt, Akt, pT202/Y204-ERK and ERK expression after trametinib treatment in Detroit 562, FaDu and SCC25 cells. The expressions were normalized to α-tubulin. All data are presented as mean ± SD from three independent experiments. The expression of all proteins in trametinib-treated samples were compared to protein expression in DMSO-treated control samples, and Student’s *t*-tests were performed for statistically significant differences in each cell line; * *p* < 0.05 trametinib-treated vs. control.

After determining the expression and phosphorylation profiles of the model cell lines, we examined the effect of the MEK inhibitor trametinib on the protein levels and relevant phosphorylations of EGFR, Akt and ERK (Figure 7). We observed a significant reduction in the phosphorylation of ERK kinases (downstream to MEK) in all three cell lines (Detroit 562 *p* = 9 × 10^−9^, FaDu *p* = 5 × 10^−8^, SCC25 *p* = 9 × 10^−9^). Interestingly, this was accompanied by a significant elevation in the levels of both pY1068-EGFR (*p* = 0.03) and pS473-Akt (*p* = 0.003) in the FaDu cells, suggesting a molecular-feedback mechanism in this cell line, which has been described previously in pancreatic- and colon-cancer cells [13,22]. This phenomenon, however, was not observed in the Detroit 562 or the SCC25 cells.

## 3. Discussion

Head-and-neck squamous cell carcinoma is the sixth most common cancer worldwide and its incidence is estimated to rise by a further 30% by 2030 [23]. The disease generally occurs in adults, with median ages at diagnosis of 66 years for HPV-negative HNSCC, 53 years for HPV-positive HNSCC and 50 years for Epstein–Barr virus (EBV)-positive HNSCC, and its prevalence is higher in men than in women [24,25]. Recent advances in biotechnology, drug development, robotic surgery, radiotherapy and immunotherapy have led to major progress in the field, particularly in the treatment of HPV-negative HNSCC. However, the outcomes of these diseases remain almost unchanged; moreover, most patients with advanced-stage HNSCC are still treated with platinum-based therapy. We believe that a better understanding of the effects of the novel therapies on cell-signaling pathways, as well as their mechanism of resistance, would help to obtain better survival rates for patients with HNSCC.

In our study, we focused mainly on kinase-inhibitor-based personalized therapy, which has already been successfully used in the treatment of HPV-negative HNSCC. We used immunohistochemistry in FFPE tumor samples to investigate the protein levels of EGFR and pY1068-EGFR. A high EGFR expression was revealed in more than 90% of the tissue samples. Interestingly, of the clinicopathological parameters, only the tumor grade showed a significant correlation with the EGFR levels, as pronounced EGFR expression was observed more frequently in the samples from tumors at advanced stages. Despite the high frequency of EGFR overexpression in the HNSCCs, inhibitory monoclonal antibodies binding to the extracellular domain of EGFR (e.g., cetuximab) and tyrosine-kinase inhibitors binding to the intracellular domain of EGFR (e.g., erlotinib, gefitinib) have not been shown to be more effective than chemo- and radiotherapy [26].

The amount of pY1068-EGFR was used as an indicator of EGFR activity. Only 10% of the tested clinical head-and-neck-tumor samples showed high EGFR activity. Nevertheless, a significant association with disease-specific survival was observed: higher EGFR activity was associated with poorer prognosis. According to this result, we hypothesize that the response to EGFR-inhibitor therapies may be more accurately predicted by the activity than by the expression of EGFR; therefore, EGFR activity may be an important prognostic marker. Our result is in line with the studies of Wheeler et al., according to which high levels of pY1068-EGFR were found to be associated with reduced progression-free survival (PFS) in tumor samples of two independent cohorts. They also found that pY1068-EGFR levels provide independent prognostic information. However, in their study, the pY1068-EGFR levels and EGFR protein expression were positively correlated with each other [6]. We employed three cell lines (Detroit 562, FaDu and SCC25) from head-and-neck cancers with diverse molecular backgrounds to model the behavior of HNSCCs.

There were no significant differences between the effects of the different EGFR inhibitors on the cell viability in the tested cell lines. The afatinib showed stronger inhibition than the erlotinib although it was not as effective as we would have expected, based on the EGFR expression. By contrast, the inhibition of MEK, a key protein in the EGFR-signaling pathway, resulted in significantly different changes in cell viability. The SCC25 was found to be much more sensitive to MEK inhibition than the other two tested cell lines, and the trametinib was more effective than the selumetinib. The effects of EGFR and MEK inhibition were also examined in combination, and a remarkable synergism was observed in all three cell lines. However, when we compared single afatinib or trametinib treatments with their combination, significant differences were only seen in the FaDu cells.

Seeking an explanation for the observed differences in sensitivity, we determined the expression and phosphorylation profiles of the cell lines. No significant differences in the expression of EGFR, Akt or ERK were found in the investigated cell lines; thus, we concluded that the basal expression levels were not responsible for the differences. However, we found significant differences in the phosphorylation levels of these proteins. The EGFR was significantly less active in the FaDu cells than in the other two cell lines. Regarding the activity of the Akt and ERK proteins, Akt kinase seemed to be more active than the ERK in the Detroit 562 cells, which was in accordance with the presence of a PIK3CA-activating mutation [14]. By contrast, the ERK kinases appeared to be more active than the Akt in the SCC25 cells. These results revealed the predominance of the PI3K/Akt pathway in the Detroit 562 cells and the predominance of the MEK/ERK pathway in the SCC25 cells, while there seemed to be no significant difference between the two investigated signaling pathways in the FaDu cells.

We also examined how trametinib treatment alters the activity of these proteins. The blockade of MEK resulted in the complete inhibition of ERK in the Detroit 562 and SCC25 cells, as expected. Interestingly, this effect was associated with markedly increased EGFR and Akt activities in the FaDu cells, which definitely indicates the operation of a feedback mechanism in these cells.

The MEK/ERK pathway is one of the best-characterized kinase cascades in cancer-cell biology. The deregulation of this pathway is frequently observed, and it plays a central role in carcinogenesis and in the progression of several tumors, including melanoma and pancreatic, lung, colorectal and breast cancers. Targeting the involved kinases may offer promising novel therapies. As many as ~18% of HNSCC patient tumors harbor MEK/ERK pathway mutations. Core pathway components (*HRAS*, *BRAF*, *MAPK1*, *RPS6KA1*) are mutated in ~10.5% of cases (TCGA HNSCC cohort) [27]. Somatic point mutations of BRAF, such as those that occur at hotspot V600E of its kinase domain, can lead to an increase in BRAF-kinase function. Mutations in this gene have been associated with various types of cancer, including non-Hodgkin lymphoma, colorectal cancer, thyroid cancer and lung carcinoma [28]. Weber et al. found BRAF mutated in 3% of HNSCC cases evaluated, while Bruckman et al., evaluating HNSCC patients, reported BRAF mutations in 2.4% of the cases. According to Carvalho et al., none of the HNSCC samples analyzed showed alterations in BRAF sequence [29,30,31]. The cell lines we investigated are BRAF wild type; thus, the BRAF protein shows normal function in the signaling pathway. Therefore, we believe that the BRAF protein does not play a role in any alterations in the ERK/CDC25/EGFR feedback loop in these cell lines. Since RAS and RAF gene mutations are relatively rare in HNSCCs, it is reasonable to inhibit the MEK/ERK signaling pathway as close to its endpoint as possible. In a thorough study, Xie et. Al. defined the mechanism of action of trametinib, and it was established that trametinib inhibits ERK in HNSCC cell lines. They found that a decrease in the expression of EGFR and Myc proteins causes a decrease in cell proliferation [32]. Our results are in line with their observation that the effectiveness of trametinib depends on the complex and complete molecular background (mutation and expression); therefore, we supplemented our study with EGFR-pathway activity (protein phosphorylation) data, investigated the known feedback mechanism and offered possible therapeutic strategies for the different tumor subtypes. However, although MEK inhibitors are currently under evaluation in clinical trials, and many have shown antitumor activity, the emergence of resistance is a critical issue in developing MEK inhibitors. Biochemical feedback loops and crosstalk with other pathways (mainly the PI3K/Akt pathway) are often responsible for resistance in tumors treated with MEK inhibitors [33,34,35].

**Figure 8:**
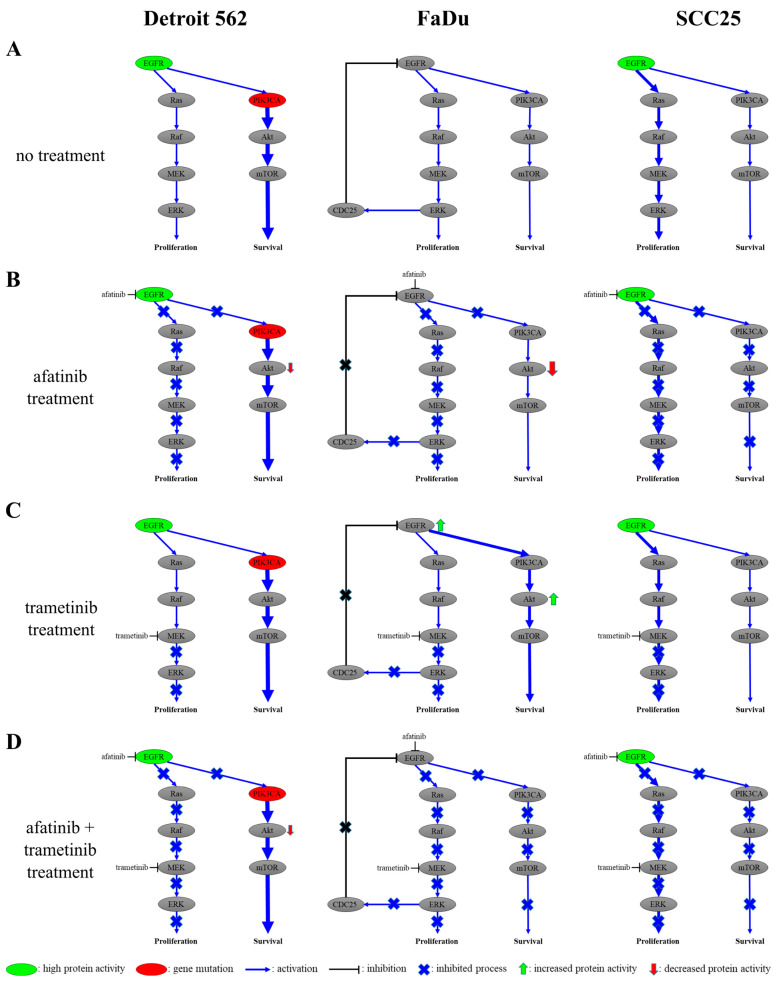
Signaling models for in vitro head-and-neck-cancer-cell lines. These models are based on our protein-expression and -phosphorylation measurements and viability assays. (**A**) The EGFR-initiated signaling in untreated cells. The PI3K/Akt pathway is significant in Detroit 562 cells due to the PIK3CA mutation, the MEK/ERK and PI3K/Akt pathways are equally strong in FaDu cells and the MEK/ERK pathway is dominant in SCC25 cells. An ERK/CDC25/EGFR feedback loop is present in FaDu cells. (**B**) Signaling under afatinib treatment. Upon treatment with afatinib, the pathways of Detroit 562 and FaDu cells are partially inhibited, while those of SCC25 cells are completely inhibited. This is due to a mutation in Detroit 562 cells and the feedback in FaDu cells. (**C**) Signaling under trametinib treatment. Upon treatment with trametinib, the pathways of Detroit 562 and FaDu cells are partially inhibited, while those of SCC25 cells are completely inhibited. This is due to a mutation in Detroit 562 cells and the feedback in FaDu cells. (**D**) Signaling under afatinib + trametinib treatment. Upon treatment with afatinib + trametinib, the pathways of Detroit 562 cells are partially inhibited, while the pathways of FaDu and SCC25 cells are completely inhibited. This is due to the PI3K mutation in Detroit 562 cells.

Based on our results, we established a theoretical model of signaling pathways in the three examined cell lines according to their genetic background (Figure 8). In normal conditions, the PI3K/Akt pathway is more active in Detroit 562 cells due to the *PIK3CA* mutation. Afatinib treatment completely inhibits the MEK/ERK pathway in these cells, whereas the activity of the PI3K/Akt pathway is reduced but not abolished. Therefore, no significant viability decrease can be achieved in Detroit 562 cells with EGFR-inhibitor treatment. Trametinib treatment only inhibits the MEK/ERK pathway; hence, the PI3K/Akt pathway remains active. Accordingly, MEK inhibitor treatment is less effective than EGFR inhibitors in this cell line. Since the afatinib treatment combined with trametinib did not augment the cell-viability reduction compared to afatinib alone, this combination is not appropriate. We assume that it would be useful to combine EGFR or MEK inhibitors with an Akt inhibitor to achieve a significant effect on Detroit 562 cells.

We saw similar activity levels in the MEK/ERK and PI3K/Akt pathways and signs of a feedback mechanism between ERK and EGFR in the FaDu cells. This type of feedback mechanism is usually used to turn off EGFR-signaling pathways [36]. Phosphorylated ERK activates a CDC25 phosphatase protein, which inactivates EGFR [13,37,38]. Accordingly, the level of pY1068-EGFR is low in this cell line, which further supports the presence of this feedback. Afatinib treatment inhibits the MEK/ERK pathway completely and the PI3K/Akt pathway only partially. Trametinib treatment completely blocks the MEK/ERK pathway in FaDu cells; thus it suspends the dephosphorylation of EGFR, as shown by increased amounts of pY1068-EGFR. The hindrance of the MEK/ERK pathway makes the PI3K/Akt pathway more active, as evidenced by the presence of elevated pS474-Akt. When FaDu cells are treated with afatinib and trametinib in combination, the MEK/ERK- and PI3K/Akt-signaling pathways are simultaneously inhibited as a dual antitumor activity. Therefore, it is advisable to use MEK and EGFR inhibitors together in FaDu cells.

Finally, in SCC25 cells, the MEK/ERK pathway is rather active in normal conditions. No mutations have been identified that affect the investigated pathways in this cell line, nor a feedback mechanism as in that seen in FaDu cells. Thus, both the MEK/ERK- and the PI3K/Akt-signaling pathway can be completely inhibited by afatinib treatment alone. Trametinib treatment resulted in the complete inhibition of the MEK/ERK pathway, which alone may be sufficient, since the PI3K/Akt pathway is less active. Both pathways are completely inhibited by treatment with afatinib + trametinib; however, this does not allow a remarkable dose reduction compared to EGFR- or MEK-inhibitor monotherapy, considering the side effects when the two agents are used concomitantly. Thus, the use of an EGFR or a MEK inhibitor alone can be an effective strategy for SCC25 cells.

## 4. Materials and Methods

### 4.1. Cell Culturing and Inhibitors

The HNSCC cell lines Detroit 562 (metastatic pharyngeal carcinoma, CCL-138™), FaDu (hypopharynx squamous cell carcinoma, HTB-43™) and SCC25 (tongue squamous cell carcinoma, CRL-1628™) were obtained from American Type Culture Collection (ATCC, Manassas, VA, USA). Detroit 562 cells were cultured in Eagle’s Minimum Essential Medium (EMEM, Lonza, Basel, Switzerland) supplemented with 10% (*V*/*V*) fetal bovine serum (FBS, Gibco, Thermo Fisher Scientific, Waltham, MA, USA), 1 mM sodium pyruvate (Lonza) and 1% (*V*/*V*) antibiotic mix (MycoZap Plus-CL, Lonza). The FaDu cells were maintained in Dulbecco’s Modified Eagle Medium (DMEM, Lonza) supplemented with 10% (*V*/*V*) FBS (Gibco), 1 mM sodium pyruvate (Lonza) and 1% (*V*/*V*) antibiotic mix (MycoZap Plus-CL, Lonza). The SCC25 cells were cultured in Dulbecco’s Modified Eagle Medium:Nutrient Mixture F-12 (DMEM:F12, Lonza) supplemented with 10% (*V*/*V*) FBS (Gibco), 400 ng/mL hydrocortisone (STEMCELL Technologies, Vancouver, BC, Canada) and 1% (*V*/*V*) antibiotic mix (MycoZap Plus-CL, Lonza) in humidified atmosphere at 37 °C and 5% CO_2_. The authentication of the cell lines was validated by STR DNA analysis (Eurofins Scientific, Luxembourg, Luxembourg). All cell lines were routinely screened for the absence of mycoplasma infection (DAPI staining) [39]. Afatinib (BIBW2992, cat. no. S1011), erlotinib HCl (OSI-744, cat. no. S1023), selumetinib (AZD6244, cat. no. S1008) and trametinib (GSK1120212, cat. no. S2673) were purchased from Selleckchem (Houston, TX, USA).

### 4.2. Cell Viability Assay and Drug Synergism

Cell-viability assay was carried out as described previously [22]. Briefly, HNSCC cells were seeded into 96-well plates at a density of 4 × 10^3^ cells/well. Cell lines were left overnight to attach, after which they were treated with decreasing concentrations of afatinib, erlotinib, selumetinib, trametinib and the combination of afatinib + trametinib (1:1) in duplicates. The final DMSO concentration was 0.2% or less. Seventy-two hours after treatment, medium was removed, 50 μL PBS containing 1 mg/mL 3-(4,5-dimethylthiaziazol-2-yl)-2,5-diphenyl-2H-tetrazolium bromide (MTT) was added to each well and cells were incubated for 1 h at 37 °C. After the incubation MTT solution was removed and tetrazolium crystals were dissolved in isopropanol containing 10% (*V*/*V*) Triton X-100 and 1% (*V*/*V*) 0.1 N HCl. Absorbance was measured at 570 nm and 690 nm with a Synergy multimode reader (BioTek, Budapest, Hungary). The 690-nanometer data were subtracted from the 570 nm for each well. Absolute IC_50_ values were calculated by non-linear regression using Graph Pad Prism 8 software (GraphPad Software, San Diego, CA, USA). Each experiment was repeated at least five times.

Potential drug synergism was confirmed and combination index (CI) at different effective doses (ED) was calculated with CompuSyn software (ComboSyn, Inc., Paramus, NJ, USA), which is based on the median-effect principle and the combination-index-isobologram Theorem [40,41]. Combination indexes were generated by CompuSyn, where CI < 0.75 indicates synergism, CI between 0.75 and 1.25 indicates additive effects and CI > 1.25 indicates antagonism [42]. In this research, combination indexes were calculated at a constant concentration ratio of the used drugs.

### 4.3. Western-Blot Analysis

Western-blot analysis was performed as previously described [43]. Cells were grown until 90% confluence in 6-well plates and were treated with 1 µM trametinib in complete medium. After treatment, cells were washed with ice-cold PBS and lysed in lysis buffer (50 mM Tris (pH 7.4), 150 mM NaCl, 1% (*V*/*V*) NP-40, 2 mM EDTA, 2 mM EGTA, 1 mM dithiothreitol, phosphatase-inhibitor cocktail (Merck, Kenilworth, NJ, USA) and protease-inhibitor cocktail (Calbiochem, Merck, Kenilworth, NJ, USA)) for 30 min on ice. Lysates were centrifuged with 13,000× *g* at 4 °C for 15 min. Protein samples of 10 μg were subjected to SDS-PAGE and electrotransferred to polyvinylidene-difluoride (PVDF) membranes. Membranes were incubated with the diluted primary antibodies at 4 °C overnight and with conjugated secondary antibodies horse-radish peroxidase (HRP) for 1 h at room temperature. The Akt (pan, clone 40D4, cat. no. 2920, dilution 1:4000), pS473-Akt (clone D9E, cat. no. 4060, dilution 1:2000), EGFR (clone D38B1, cat. no. 4267, dilution 1:2000), pY1068-EGFR (clone D7A5, cat. no. 3777, dilution 1:1000), ERK1/2 (clone 3A7, cat. no. 9107, dilution: 1:4000), pT202/Y204-ERK1/2 (clone D13.14.4E, cat. no. 4370, dilution 1:2000) monoclonal antibodies were purchased from Cell Signaling Technology (Danvers, MA, USA) and α-tubulin (clone DM1A, cat. no. T9026, dilution 1:40,000) monoclonal antibody was purchased from Merck Millipore (Burlington, MA, USA). Secondary antibodies of Anti-mouse IgG (cat. no. 7076, dilution 1:8000) and Anti-rabbit IgG (cat. no. 7054, dilution 1:2000) were purchased from Cell Signaling Technology. Bands were visualized by enhanced chemiluminescence (ECL) detection system (Perkin Elmer, Waltham, MA, USA) and quantified by ImageJ v1.48 software (NIH, Bethesda, MD, USA). Every experiment was carried out at least three times. The original images can be found in Supplementary Materials (Figures S1 and S2).

### 4.4. Patients

Altogether, 97 therapy-naive patients were who were diagnosed with squamous cell carcinoma of the oropharynx, hypopharynx and larynx at the Department of Oto-Rhino-Laryngology and Head and Neck Surgery, Semmelweis University between 2012 and 2014 were enrolled consecutively. All subjects gave their informed consent for inclusion before they participated in the study. The study was conducted in accordance with the Declaration of Helsinki, and the protocol was approved by Semmelweis University’s Regional, Institutional Scientific and Research Ethics Committee (ethical-license no.: 105/2014). The most important characteristics of our cohort are shown in Table 1.

### 4.5. Tissue Microarray (TMA) and Immunohistochemistry

Tissue-microarray blocks containing 2-mm-diameter cores of FFPE tissue samples were prepared using the TMA Master tool (3DHISTECH Kft, Budapest, Hungary). Tissue sections (4 μm) were cut on adhesion slides and were stained with hematoxylin and eosin. The EGFR and pY1068-EGFR antibodies used for immunohistochemistry are the same as those used for Western blot. The staining-method protocol was carried out as described previously [44].

Immunohistochemistry was performed in TMA sections following routine dewaxing and rehydration. For antigen retrieval, the samples were boiled in Tris-EDTA buffer solution (pH 9.0) for 58 min. Endogenous peroxidase activity was blocked using 3% hydrogen peroxide in methanol for 15 min. Immunostaining included the following sequential incubation steps: usage of 3% BSA in 0.1 M Tris-buffered saline with Tween^®^ 20 pH 7.4 (TBST) as a protein block, for 15 min; application of the optimally diluted primary antibody (1:400), overnight (16 h); and use of the HISTOLS-MR-T HRP polymer reagent for 40 min. Samples were washed after each incubation step for 10 min in TBST. Peroxidase activity was visualized using DAB Quanto (Thermo Fisher Scientific) for 5 min under microscopic control. Finally, nuclear counterstaining was applied using hematoxylin and eosin. All incubations were performed in humidity chambers at room temperature. Immunostained slides were digitalized, applying a Pannoramic Scan instrument (3DHISTECH Kft, Budapest, Hungary). The histologic evaluation and the scoring of immunoreactions were performed by 2 independent assessors using the Pannoramic Viewer software (3DHISTECH Kft, Budapest, Hungary).

An alternative 3-grade scoring approach was used for the evaluation of EGFR expression and pY1068-EGFR protein phosphorylation, applied in several earlier studies for the evaluation of EGFR protein expression [45,46]. Briefly, the percentage of stained cells was multiplied by the grade intensity of staining (in four grades: 1, negative; 2, weak; 3, moderate; 4, intense) which results in a value between 0 and 400. Cores with scores 0 (0), 1 to 200 (1), 201 to 300 (2) and 301 to 400 (3) were referred to as negative, low, intermediate, or high protein expression, respectively. For statistical analysis, scores were dichotomized along different threshold values. The most reproducible threshold for assessment was set up when score 1 was considered as low/negative EGFR expression, whereas scores 2 and 3 were taken as high/positive EGFR expression. For pY1068-EGFR phosphorylation, scores 0, 1 and 2 were considered as low/negative protein phosphorylation, whereas score 3 was taken as high/positive pY1068-EGFR phosphorylation.

### 4.6. Statistical Analysis

Statistica 13 (TIBCO Software Inc., Palo Alto, CA, USA) and Graph Pad Prism 8 (GraphPad Software) software were used to carry out the statistical analysis; measured values are indicated as mean ± standard deviation (SD). Student’s *t*-test and multiple *t*-tests with Bonferroni–Sidak correction were used to compare groups. Two-sided test was selected. A *p* < 0.05 value was considered as statistically significant. The cell viability was compared in samples treated with control and EGFR inhibitors (afatinib, erlotinib), MEK inhibitors (selumetinib, trametinib) and afatinib + trametinib at all concentrations used, and this method was applied in each cell line. We determined and compared the IC_50_ value for each inhibitor and each studied cell line (Tables S1–S11). The Akt, pS473-Akt, EGFR, pY1068-EGFR, ERK1/2 and pT202/Y204-ERK1/2 protein levels of HNSCC cell lines were compared to each other (Tables S12 and S13). When the effects of trametinib on the protein expression of HNSCC cell lines were investigated, the expression pattern of all proteins in DMSO-treated control samples was compared to that of the trametinib-treated cell samples. This method was applied in each cell line (Tables S14 and S15).

Statistical analysis of patient data was performed using IBM SPSS Statistics for Mac version 27.0.0 (SPSS Inc., Chicago, IL, USA). The Pearson χ^2^ tests and the Fisher’s exact tests were used to test correlations between discrete variables. In case of survival analysis, Kaplan–Meier estimation with log-rank test as well as univariate and multivariate regression were applied. All tests were two-sided and *p*-values < 0.05 were considered statistically significant. Tumor localization, tumor size, lymph-node metastasis, distant metastasis, stage, grade, smoking, alcohol consumption and the biomarkers listed above were used in the analysis (Table S16).

## 5. Conclusions

The great efforts to widen the therapeutic and diagnostic possibilities and the remarkable scientific progress in the field of HNSCC have only modestly improved the survival rates of the disease over the past three decades. Our results clearly demonstrate the high versatility of this cancer type, and they highlight the importance of personalized diagnosis and treatment. The presented findings also show that, in addition to the analysis of EGFR-expression levels, it is essential to investigate the phosphorylation state of the EGFR protein. Therefore, further studies are needed to understand the underlying pathomechanism of this cancer type and to find new modalities in the treatment of HNSCC.

## Figures and Tables

**Table 1:** Patient characteristics at time of diagnosis.

Variable	No. of Patients
Total no. of patients	97
Sex	
Male	79
Female	18
Age (year)	
Mean	61 (43–81)
Localization	
Oropharynx	36
Hypopharynx	35
Supraglottis	24
Glottis	2
TNM ^1^ T parameter	
1	14
2	29
3	27
4a	18
4b	9
TNM ^1^ N parameter	
0	46
1	16
2a	6
2b	12
2c	14
3	3
TNM ^1^ M parameter	
0	92
1	5
TNM ^1^ stage	
1	26
2	66
3	5
Grade	
1	6
2	43
3	34
No data	14
Tobacco use	
Never	10
Previously yes	28
Currently	57
No data	2
Alcohol use	
Never	23
Previously yes	32
Currently	42

^1^ TNM: tumor, node and metastasis, UICC TNM 7th edition.

## Data Availability

Not applicable.

## Supplementary Materials

The following supporting information can be downloaded at: https://www.mdpi.com/article/10.3390/ijms24032782/s1.
