# Supplementary material for: MEK Is a Potential Indirect Target in Subtypes of Head and Neck Cancers
Source: Int J Mol Sci. 2023 Feb 1;24(3):2782. doi: 10.3390/ijms24032782 (PMC9917750; doi:10.3390/ijms24032782)
Supplement: Supplementary file 1 [file ijms-24-02782-s001.zip › ijms-2084919-supplementary.pdf]

International Journal of *Molecular Sciences*

MDPI

Supplementary Materials

MEK Is a Potential Indirect Target in Subtypes of Head and Neck Cancers

Bianka Gurbi, Diána Brauswetter, Kinga Pénzes, Attila Varga, Tibor Krenács, Kornél Dános, Ede Birtalan, László Tamás and Miklós Csala

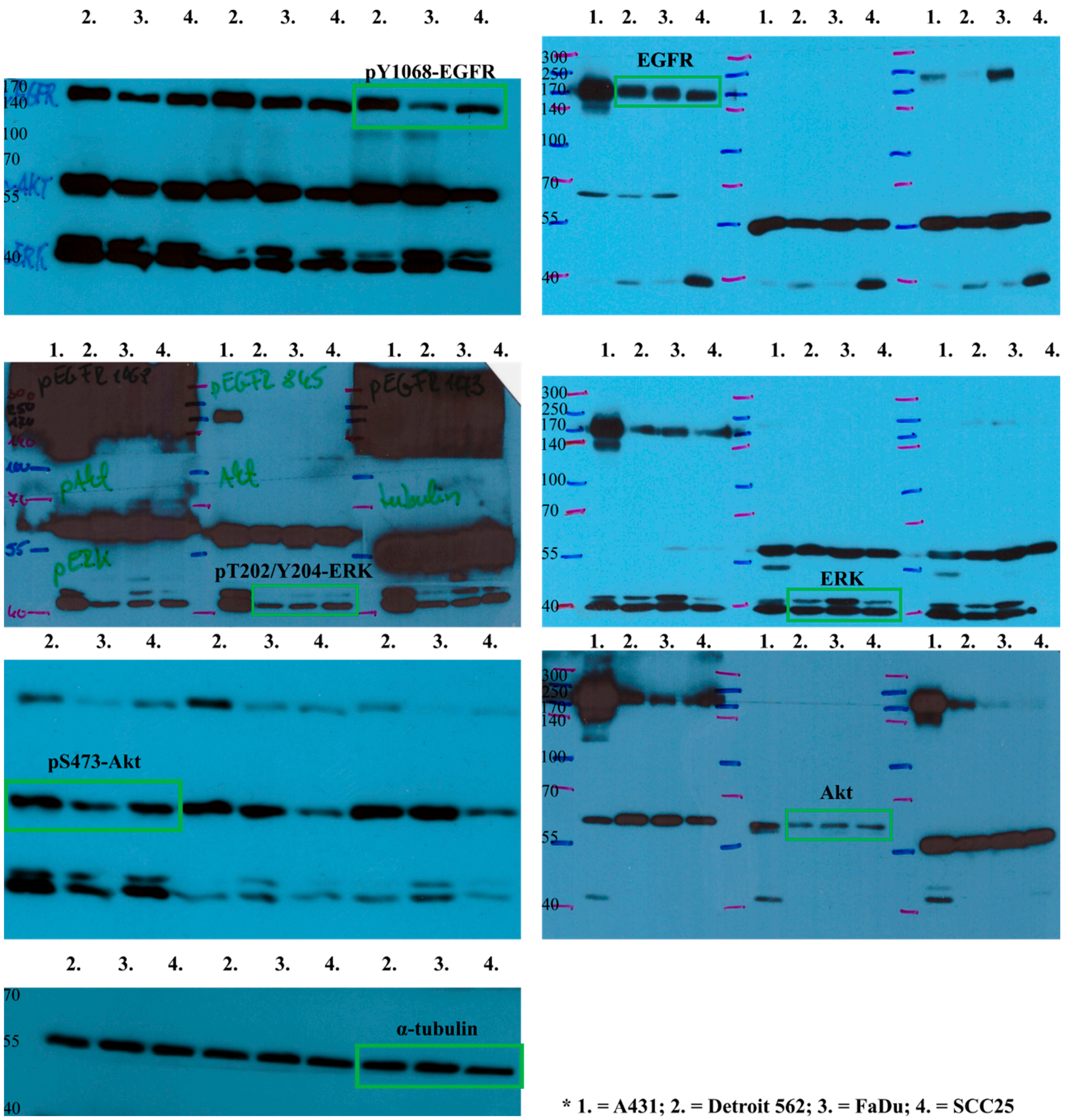

Figure S1. Original images of western blot analysis: protein expression and phosphorylation in head and neck squamous cell carcinoma (HNSCC) cell lines (Detroit 562, FaDu and SCC25) and human skin epidermoid carcinoma cell line, A431. We were not used A431 in this article. Cells were subjected to western blot analysis with antibodies against pY1068-EGFR, EGFR, pS473-Akt, Akt, pT202/Y204-ERK, ERK and the loading control, α-tubulin. (Figure 6A).

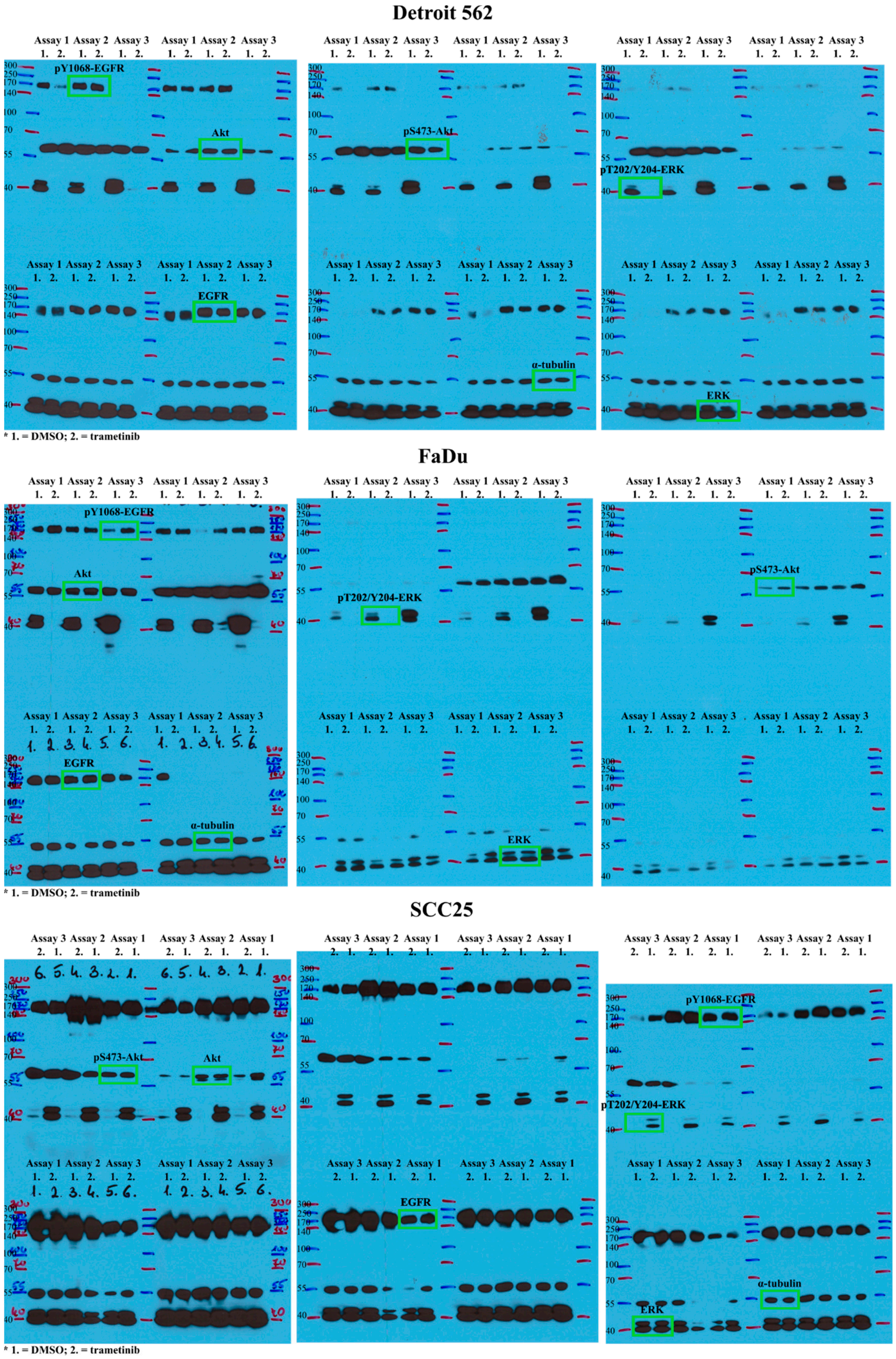

Figure S2. Original images of western blot analysis: changes of protein expression and phosphorylation after trametinib treatment in head and neck squamous cell carcinoma (HNSCC) cell lines (Detroit 562, FaDu and SCC25). Treated cells were subjected to western blot analysis with antibodies against pY1068-EGFR, EGFR, pS473-Akt, Akt, pT202/Y204-ERK, ERK and the loading control, α-tubulin. (Figure 7A)

Table S1. Effects of EGFR inhibitors on cell viability – numerical data. HNSCC cell lines were analyzed in parallel by MTT after 72 h of treatment with EGFR inhibitors different concentrations. (Figure 3A,B,C,D).

Concentration (μM)	Effect of afatinib on Detroit 562 cell viability (%)						
	Sample 1	Sample 2	Sample 3	Sample 4	Sample 5	Mean	SD
5.00000	25.57692	31.97787	43.43017	20.85071	40.81226	32.52959	9.64968
1.66667	61.05478	38.15932	54.47165	53.26575	51.18763	51.62783	8.38499
0.55556	66.00816	45.09456	54.65191	54.88247	48.80098	53.88762	7.93210
0.18519	67.13869	48.63582	54.28867	55.70214	48.26413	54.80589	7.64907
0.06173	69.84266	50.10948	54.46893	57.45465	50.34746	56.44464	8.08919
0.02058	67.16200	56.06712	55.94095	61.34769	59.41558	59.98667	4.62046
0.00686	67.90210	54.55955	58.34419	62.78171	71.84438	63.08639	6.98538
0.00229	65.64685	61.73895	67.34839	67.56232	98.06904	72.07311	14.71861
0.00076	68.31585	62.23307	76.39066	72.47874	96.52825	75.18931	13.03121
0.00025	70.23310	70.98593	82.86844	81.72147	103.37349	81.83649	13.39117
Concentration (μM)	Effect of erlotinib on Detroit 562 cell viability (%)						
	Sample 1	Sample 2	Sample 3	Sample 4	Sample 5	Mean	SD
5.00000	37.47073	46.52655	44.98392	46.45666	37.38342	42.56426	4.72997
1.66667	48.66402	60.36747	60.56622	67.41156	54.01741	58.20534	7.13405
0.55556	71.99935	71.51898	69.54322	78.19870	67.45660	71.74337	4.03126
0.18519	80.08859	79.77743	78.06099	86.80373	80.36013	81.01818	3.35612
0.06173	82.73515	92.17567	93.47057	97.71487	91.90062	91.59938	5.47490
0.02058	94.54205	106.02619	97.84327	102.32099	99.77522	100.10154	4.36296
0.00686	95.02896	110.56940	106.00297	107.70271	106.96710	105.25423	5.96441
0.00229	106.99853	117.77900	106.61794	111.31473	111.75092	110.89222	4.52144
0.00076	102.46101	114.49212	107.22459	112.97441	116.83007	110.79644	5.85328
0.00025	101.55801	113.65073	97.74210	104.50036	102.44871	103.97998	5.93540
Concentration (μM)	Effect of afatinib on FaDu cell viability (%)						
	Sample 1	Sample 2	Sample 3	Sample 4	Sample 5	Mean	SD
5.00000	4.61660	4.64419	4.47484	5.87177	7.68369	5.45822	1.36581
1.66667	44.44075	39.82736	35.33959	45.09049	45.37238	42.01411	4.35780
0.55556	54.23582	48.54068	47.06849	53.02803	55.77718	51.73004	3.74984
0.18519	55.68096	51.49959	46.91631	55.16553	56.01209	53.05490	3.87934
0.06173	54.71736	49.85580	45.06398	55.63783	46.43277	50.34155	4.75830
0.02058	55.03877	49.85566	45.21759	54.69543	52.90708	51.54291	4.08892
0.00686	56.48340	50.18461	45.83392	58.86339	49.66829	52.20672	5.33260
0.00229	61.46203	52.32263	46.60529	59.26175	58.69839	55.67002	6.10314
0.00076	65.47680	61.85778	52.62930	56.72368	69.41750	61.22101	6.70342
0.00025	71.41800	69.42006	59.72427	65.53299	85.45809	70.31068	9.57029
Concentration (μM)	Effect of erlotinib on FaDu cell viability (%)						
	Sample 1	Sample 2	Sample 3	Sample 4	Sample 5	Mean	SD
5.00000	34.18449	31.41341	33.45474	39.41195	34.58126	34.60917	2.94930
1.66667	48.60963	48.66445	50.35047	61.05869	48.13018	51.36268	5.48511
0.55556	62.80080	59.46002	59.05405	69.93896	66.35987	63.52274	4.64256
0.18519	81.87166	83.38455	70.16923	81.46629	79.84245	79.34684	5.28309
0.06173	104.84626	93.45822	82.03559	93.35568	82.97678	91.33451	9.32046
0.02058	105.12032	99.31443	95.67726	104.31897	97.84411	100.45502	4.11218
0.00686	106.81484	101.65241	94.74702	111.34271	94.54395	101.82018	7.39301
0.00229	108.26537	103.85421	93.70106	111.09171	94.19569	102.22161	7.98258
0.00076	96.68115	101.27564	99.73403	115.29528	94.76368	101.54996	8.09422
0.00025	105.57487	103.76125	98.57059	116.97003	97.84411	104.54417	7.69175
Concentration (μM)	Effect of afatinib on SCC25 cell viability (%)						
	Sample 1	Sample 2	Sample 3	Sample 4	Sample 5	Mean	SD
5.00000	28.00276	14.73113	27.00042	30.80895	22.42084	24.59282	6.28560
1.66667	50.73605	39.71254	31.76637	46.03316	34.60144	40.56991	7.85903
0.55556	53.45040	44.90191	31.01064	45.05243	32.87465	41.45801	9.37309

0.18519	50.94935	43.34969	28.40300	42.84630	31.93900	39.49747	9.18446
0.06173	55.67055	44.90191	29.91448	41.55598	30.22250	40.45308	10.82020
0.02058	55.62999	45.62393	30.64977	41.23340	30.99992	40.82740	10.51727
0.00686	58.04993	44.90191	32.04547	48.68970	35.98777	43.93496	10.33553
0.00229	70.95476	55.89448	40.46746	50.38051	42.40310	52.02006	12.26958
0.00076	80.89137	58.08759	53.07655	54.52212	50.04654	59.32483	12.39798
0.00025	91.85994	68.46631	64.46016	71.17747	61.31892	71.45656	12.01082

Concentration (μM)	Effect of erlotinib on SCC25 cell viability (%)						
	Sample 1	Sample 2	Sample 3	Sample 4	Sample 5	Mean	SD
5.00000	45.20697	46.20106	38.67439	37.78785	27.10792	38.99564	7.63837
1.66667	56.96805	57.01091	48.21531	50.22475	38.13929	50.11166	7.77104
0.55556	67.37836	59.29126	58.32982	61.76265	49.13876	59.18017	6.62278
0.18519	73.95062	65.79068	76.25459	66.29454	59.73153	68.40439	6.68745
0.06173	95.51561	84.22422	102.83913	81.49755	77.54652	88.32461	10.51155
0.02058	107.24764	87.69243	116.07687	90.63502	91.45136	98.62066	12.38688
0.00686	107.34931	94.84735	126.92912	95.45890	91.37161	103.19126	14.57643
0.00229	103.51489	107.06354	129.19193	100.96809	97.11590	107.57087	12.62024
0.00076	97.39651	91.81614	112.52888	91.19598	93.56459	97.30042	8.84892
0.00025	103.92157	105.12484	108.67065	96.72820	94.37002	101.76306	5.99338

Table S2. Statistical analysis (Multiple t-tests) of effects of EGFR inhibitors on cell viability. HNSCC cell lines were analyzed in parallel by MTT after 72 h of treatment with EGFR inhibitors different concentrations. Statistical analysis was performed by Multiple t-tests. The cell viability in all concentration were compared to each other in each cell line. Statistical significance determined using the Bonferroni-Sidak method, with alpha = 0.05. Computations assume that all rows are sample from populations with the same scatter (SD). Number of t tests: 10. Red color indicates if $p < 0.05$. (Figure 3A,B,C).

Concentration (μM)	Multiple t-tests on Detroit 562							
	P value	Mean of afatinib	Mean of erlotinib	Difference	SE of difference	t ratio	df	Adjusted P Value
5.00000	0.049141	32.53	42.56	-10.03	5.023	1.998	80.00	0.395827
1.66667	0.194117	51.63	58.21	-6.578	5.023	1.309	80.00	0.884463
0.55556	0.000638	53.89	71.74	-17.86	5.023	3.555	80.00	0.006365
0.18519	0.000001	54.81	81.02	-26.21	5.023	5.219	80.00	0.000014
0.06173	<0.000001	56.44	91.60	-35.15	5.023	6.999	80.00	<0.000001
0.02058	<0.000001	59.99	100.1	-40.11	5.023	7.986	80.00	<0.000001
0.00686	<0.000001	63.09	105.3	-42.17	5.023	8.395	80.00	<0.000001
0.00229	<0.000001	72.07	110.9	-38.82	5.023	7.728	80.00	<0.000001
0.00076	<0.000001	75.19	110.8	-35.61	5.023	7.089	80.00	<0.000001
0.00025	0.000032	81.84	104.0	-22.14	5.023	4.408	80.00	0.000321

Concentration (μM)	Multiple t-tests on FaDu							
	P value	Mean of afatinib	Mean of erlotinib	Difference	SE of difference	t ratio	df	Adjusted P Value
5.00000	<0.000001	5.458	34.61	-29.15	3.812	7.647	80.00	<0.000001
1.66667	0.016371	42.01	51.36	-9.349	3.812	2.452	80.00	0.152161
0.55556	0.002725	51.73	63.52	-11.79	3.812	3.093	80.00	0.026916
0.18519	<0.000001	53.05	79.35	-26.29	3.812	6.897	80.00	<0.000001
0.06173	<0.000001	50.34	91.33	-40.99	3.812	10.75	80.00	<0.000001
0.02058	<0.000001	51.54	100.5	-48.91	3.812	12.83	80.00	<0.000001
0.00686	<0.000001	52.21	101.8	-49.61	3.812	13.01	80.00	<0.000001
0.00229	<0.000001	55.67	102.2	-46.55	3.812	12.21	80.00	<0.000001
0.00076	<0.000001	61.22	101.5	-40.33	3.812	10.58	80.00	<0.000001
0.00025	<0.000001	70.31	104.5	-34.23	3.812	8.980	80.00	<0.000001

Concentration (μM)	Multiple t-tests on SCC25							
	P value	Mean of afatinib	Mean of erlotinib	Difference	SE of difference	t ratio	df	Adjusted P Value
5.00000	0.025929	24.59	39.00	-14.40	6.346	2.270	80.00	0.231037
1.66667	0.136639	40.57	50.11	-9.542	6.346	1.504	80.00	0.769895

0.55556	0.006539	41.46	59.18	-17.72	6.346	2.793	80.00	0.063503
0.18519	0.000019	39.50	68.40	-28.91	6.346	4.555	80.00	0.000185
0.06173	<0.000001	40.45	88.32	-47.87	6.346	7.543	80.00	<0.000001
0.02058	<0.000001	40.83	98.62	-57.79	6.346	9.107	80.00	<0.000001
0.00686	<0.000001	43.93	103.2	-59.26	6.346	9.337	80.00	<0.000001
0.00229	<0.000001	52.02	107.6	-55.55	6.346	8.753	80.00	<0.000001
0.00076	<0.000001	59.32	97.30	-37.98	6.346	5.984	80.00	<0.000001
0.00025	0.000008	71.46	101.8	-30.31	6.346	4.776	80.00	0.000080

Table S3. Statistical analysis (Multiple t-tests) of effect of afatinib on cell viability. HNSCC cell lines were analyzed in parallel by MTT after 72 h of treatment with afatinib different concentrations. Statistical analysis was performed by Multiple t-tests. The cell viability in all concentration were compared to each other. Statistical significance determined using the Bonferroni-Sidak method, with alpha = 0.05. Computations assume that all rows are sample from populations with the same scatter (SD). Number of t tests: 10. Red color indicates if $p < 0.05$. (Figure 3D).

Concentration (µM)	Multiple t-tests							
	P value	Mean of Detroit 562	Mean of FaDu	Difference	SE of difference	t ratio	df	Adjusted P Value
5.00000	<0.000001	32.53	5.458	27.07	5.055	5.355	80.00	0.000008
1.66667	0.060803	51.63	42.01	9.614	5.055	1.902	80.00	0.465968
0.55556	0.670668	53.89	51.73	2.158	5.055	0.4268	80.00	0.999985
0.18519	0.729969	54.81	53.05	1.751	5.055	0.3464	80.00	0.999998
0.06173	0.230876	56.44	50.34	6.103	5.055	1.207	80.00	0.927563
0.02058	0.098765	59.99	51.54	8.444	5.055	1.670	80.00	0.646505
0.00686	0.034398	63.09	52.21	10.88	5.055	2.152	80.00	0.295333
0.00229	0.001717	72.07	55.67	16.40	5.055	3.245	80.00	0.017034
0.00076	0.007101	75.19	61.22	13.97	5.055	2.763	80.00	0.068781
0.00025	0.025270	81.84	70.31	11.53	5.055	2.280	80.00	0.225820

Concentration (µM)	Multiple t-tests							
	P value	Mean of Detroit 562	Mean of SCC25	Difference	SE of difference	t ratio	df	Adjusted P Value
5.00000	0.217967	32.53	24.59	7.937	6.392	1.242	80.00	0.914444
1.66667	0.087480	51.63	40.57	11.06	6.392	1.730	80.00	0.599666
0.55556	0.055333	53.89	41.46	12.43	6.392	1.945	80.00	0.434036
0.18519	0.018956	54.81	39.50	15.31	6.392	2.395	80.00	0.174182
0.06173	0.014392	56.44	40.45	15.99	6.392	2.502	80.00	0.134946
0.02058	0.003625	59.99	40.83	19.16	6.392	2.998	80.00	0.035663
0.00686	0.003638	63.09	43.93	19.15	6.392	2.996	80.00	0.035790
0.00229	0.002387	72.07	52.02	20.05	6.392	3.137	80.00	0.023611
0.00076	0.015158	75.19	59.32	15.86	6.392	2.482	80.00	0.141644
0.00025	0.108318	81.84	71.46	10.38	6.392	1.624	80.00	0.682239

Concentration (µM)	Multiple t-tests							
	P value	Mean of FaDu	Mean of SCC25	Difference	SE of difference	t ratio	df	Adjusted P Value
5.00000	0.000414	5.458	24.59	-19.13	5.192	3.685	80.00	0.004136
1.66667	0.781624	42.01	40.57	1.444	5.192	0.2781	80.00	>0.999999
0.55556	0.051339	51.73	41.46	10.27	5.192	1.978	80.00	0.409647
0.18519	0.010776	53.05	39.50	13.56	5.192	2.611	80.00	0.102677
0.06173	0.060451	50.34	40.45	9.888	5.192	1.904	80.00	0.463963
0.02058	0.042289	51.54	40.83	10.72	5.192	2.064	80.00	0.350850
0.00686	0.115091	52.21	43.93	8.272	5.192	1.593	80.00	0.705569
0.00229	0.484132	55.67	52.02	3.650	5.192	0.7029	80.00	0.998665
0.00076	0.715938	61.22	59.32	1.896	5.192	0.3652	80.00	0.999997
0.00025	0.825901	70.31	71.46	-1.146	5.192	0.2207	80.00	>0.999999

Table S4. Statistical analysis (Student's t-test) of effects of EGFR inhibitors on cell viability. HNSCC cell lines were analyzed in parallel by MTT after 72 h of treatment with EGFR inhibitors different concentrations. Statistical analysis was performed by Student's t-test, the IC_{50} concentrations of the EGFR inhibitors were compared to each other in each cell line. Red color indicates if $p < 0.05$. (Figure 3E).

Group 1 vs. Group 2	T-test for Independent Samples (EGFR_inhibitors_IC50) Note: Variables were treated as independent samples										
	Mean Group 1	Mean Group 2	t-value	df	p	Valid N Group 1	Valid N Group 2	Std.Dev. Group 1	Std.Dev. Group 2	F-ratio Variances	p Variances
Detroit 562 afatinib IC50 vs. Detroit 562 erlotinib IC50	0.425565	2.826000	-5.17866	7	0.001283	4	5	0.339693	0.865450	6.4910	0.156315
FaDu afatinib IC50 vs. FaDu erlotinib IC50	0.042756	1.718200	-7.64343	8	0.000061	5	5	0.028749	0.489303	289.6811	0.000071
SCC25 afatinib IC50 vs. SCC25 erlotinib IC50	0.058344	1.745860	-4.64236	8	0.001661	5	5	0.116778	0.804386	47.4471	0.002521
Detroit 562 afatinib IC50 vs. FaDu afatinib IC50	0.425565	0.042756	2.55396	7	0.037884	4	5	0.339693	0.028749	139.6165	0.000335
Detroit 562 afatinib IC50 vs. FaDu erlotinib IC50	0.425565	1.718200	-4.46484	7	0.002919	4	5	0.339693	0.489303	2.0748	0.575059
Detroit 562 afatinib IC50 vs. SCC25 afatinib IC50	0.425565	0.058344	2.28796	7	0.055975	4	5	0.339693	0.116778	8.4616	0.066249
Detroit 562 afatinib IC50 vs. SCC25 erlotinib IC50	0.425565	1.745860	-3.03990	7	0.018849	4	5	0.339693	0.804386	5.6073	0.188263
Detroit 562 erlotinib IC50 vs. FaDu erlotinib IC50	2.826000	1.718200	2.49158	8	0.037430	5	5	0.865450	0.489303	3.1284	0.295183
Detroit 562 erlotinib IC50 vs. SCC25 erlotinib IC50	2.826000	1.745860	2.04416	8	0.075186	5	5	0.865450	0.804386	1.1576	0.890635
FaDu afatinib IC50 vs. SCC25 afatinib IC50	0.042756	0.058344	-0.28982	8	0.779322	5	5	0.028749	0.116778	16.5000	0.018845
FaDu afatinib IC50 vs. SCC25 erlotinib IC50	0.042756	1.745860	-4.73134	8	0.001480	5	5	0.028749	0.804386	782.8768	0.000010
FaDu erlotinib IC50 vs. SCC25 erlotinib IC50	1.718200	1.745860	-0.06569	8	0.949235	5	5	0.489303	0.804386	2.7025	0.358868

Table S5. Effects of MEK inhibitors on cell viability – numerical data. HNSCC cell lines were analyzed in parallel by MTT after 72 h of treatment with MEK inhibitors different concentrations. (Figure 4A,B,C,D).

Concentration (μM)	Effect of selumetinib on Detroit 562 cell viability (%)						
	Sample 1	Sample 2	Sample 3	Sample 4	Sample 5	Mean	SD
5.00000	73.65024	64.86134	61.67514	58.08456	73.67914	66.39008	7.06027
1.66667	76.39812	74.68071	66.98641	77.69146	75.63460	74.27826	4.22245
0.55556	80.65174	78.49427	72.68127	76.05506	79.13112	77.40269	3.11622
0.18519	91.58031	86.17809	76.19504	82.77639	82.71183	83.88833	5.61731
0.06173	86.61172	90.19754	81.75711	92.26473	84.96763	87.15974	4.17083
0.02058	92.87295	97.27461	86.28446	88.27215	91.76489	91.29381	4.26349
0.00686	99.58575	100.21783	93.55981	98.82463	101.30488	98.69858	3.01292
0.00229	103.02376	103.54400	98.83472	102.67297	96.54877	100.92485	3.07571
0.00076	108.36929	109.22953	95.91682	106.96364	97.66567	103.62899	6.32446
0.00025	104.83066	105.79394	99.08899	107.45642	92.29845	101.89369	6.21926
Concentration (μM)	Effect of trametinib on Detroit 562 cell viability (%)						
	Sample 1	Sample 2	Sample 3	Sample 4	Sample 5	Mean	SD
5.00000	54.92266	52.46894	58.69705	62.26100	55.59690	56.78931	3.77990
1.66667	55.15139	47.89731	74.30339	74.84671	63.67028	63.17382	11.81208
0.55556	61.50387	56.11176	71.86504	72.97958	62.79914	65.05188	7.19125
0.18519	59.43209	58.90356	73.80777	68.71908	66.34818	65.44213	6.33279
0.06173	59.92484	62.87955	74.68497	71.97104	61.91312	66.27470	6.59644
0.02058	63.05272	70.08911	73.96637	73.44660	69.52117	70.01520	4.36211
0.00686	67.30068	79.83779	75.56218	67.60167	67.85530	71.63152	5.74562
0.00229	78.79504	79.81235	75.90910	74.46087	73.29047	76.45357	2.78551
0.00076	70.15534	80.36420	77.12828	74.95272	76.19856	75.75982	3.71911
0.00025	85.24166	82.94294	76.27583	68.50706	76.43866	77.88123	6.56373
Concentration (μM)	Effect of selumetinib on FaDu cell viability (%)						
	Sample 1	Sample 2	Sample 3	Sample 4	Sample 5	Mean	SD
5.00000	58.02224	58.09993	68.72781	70.84017	63.61042	63.86012	5.91055
1.66667	65.80772	66.80704	66.84911	77.04587	74.62443	70.22683	5.20746
0.55556	74.97569	75.40796	79.27515	86.97552	81.36127	79.59912	4.91333
0.18519	81.44210	83.29733	87.52959	95.01183	84.03590	86.26335	5.36523
0.06173	95.56716	87.81476	81.89349	101.89500	86.36828	90.70774	7.96335
0.02058	93.56530	90.47210	88.06213	96.93595	77.84925	89.37695	7.25426
0.00686	99.49901	93.98391	88.60947	99.41281	84.22774	93.14659	6.71661
0.00229	101.66786	102.13464	89.24556	87.86352	84.04996	92.99231	8.35398
0.00076	105.12409	95.17530	91.07988	95.28263	79.35279	93.20294	9.30944
0.00025	105.55321	100.30727	92.50000	124.89050	83.81708	101.41361	15.48075
Concentration (μM)	Effect of trametinib on FaDu cell viability (%)						
	Sample 1	Sample 2	Sample 3	Sample 4	Sample 5	Mean	SD
5.00000	55.06543	48.80327	50.53982	51.36895	63.46816	53.84913	5.84313
1.66667	55.03323	52.81018	55.85263	54.69510	56.91695	55.06162	1.52251
0.55556	57.91698	53.06690	53.42094	56.64600	65.34352	57.27887	4.96162
0.18519	58.03573	53.08164	55.55610	57.99149	70.65404	59.06380	6.79394
0.06173	65.01869	55.08509	56.31570	54.87554	64.31799	59.12260	5.09837
0.02058	73.32583	56.07945	57.37183	55.66368	67.31569	61.95130	7.95520
0.00686	69.54857	59.49920	61.20879	59.37132	72.21027	64.36763	6.06202
0.00229	75.99425	64.38438	64.34311	66.19719	81.67312	70.51841	7.88743
0.00076	72.77643	67.64874	71.44440	67.56328	79.80493	71.84756	5.00825
0.00025	74.48695	71.66056	67.52759	69.71293	81.40419	72.95844	5.37073
Concentration (μM)	Effect of selumetinib on SCC25 cell viability (%)						
	Sample 1	Sample 2	Sample 3	Sample 4	Sample 5	Mean	SD
5.00000	58.01604	51.93568	36.47158	60.17339	39.86382	49.29210	10.66251
1.66667	73.98165	84.14348	46.35194	72.75074	50.95788	65.63714	16.20276

0.55556	76.57791	93.41991	53.00515	76.36970	59.49900	71.77434	15.93494
0.18519	88.38865	95.67100	62.81286	85.03310	63.73024	79.12717	14.97978
0.06173	94.60810	101.68213	83.61323	86.46845	74.53928	88.18224	10.41011
0.02058	104.42072	116.72233	93.68153	97.56098	82.56176	98.98946	12.68894
0.00686	105.79710	103.04267	94.29457	100.18616	88.90594	98.44529	6.82721
0.00229	108.52628	94.08782	110.69380	118.02378	98.27528	105.92139	9.67633
0.00076	99.01595	95.67100	100.21405	101.09091	94.89029	98.17644	2.75803
0.00025	99.86708	111.20594	89.60672	105.72163	107.11063	102.70240	8.37249

Concentration (µM)	Effect of trametinib on SCC25 cell viability (%)						
	Sample 1	Sample 2	Sample 3	Sample 4	Sample 5	Mean	SD
5.00000	38.03687	32.24167	31.33205	36.52847	22.63963	32.15574	6.01977
1.66667	40.68203	27.34801	33.16313	37.80532	23.58586	32.51687	7.09815
0.55556	38.89401	29.94542	30.46429	38.08667	25.90018	32.65811	5.61687
0.18519	41.04147	32.46753	35.15541	39.10579	23.58586	34.27121	6.84510
0.06173	44.80184	39.73273	36.16313	41.27393	29.71628	38.33758	5.73172
0.02058	55.88940	49.97177	42.51641	46.80440	36.00639	46.23767	7.51578
0.00686	65.29954	54.07491	49.79633	51.31575	38.14542	51.72639	9.72217
0.00229	73.17051	67.70186	59.25290	56.62392	41.96673	59.74318	11.93321
0.00076	73.88940	66.61020	59.88514	56.50582	46.83155	60.74442	10.24048
0.00025	79.85253	75.83286	59.21622	59.42354	49.32115	64.72926	12.72750

Table S6. Statistical analysis (Multiple t-tests) of effects of MEK inhibitors on cell viability. HNSCC cell lines were analyzed in parallel by MTT after 72 h of treatment with MEK inhibitors different concentrations. Statistical analysis was performed by Multiple t-tests. The cell viability in all concentration were compared to each other in each cell line. Statistical significance determined using the Bonferroni-Sidak method, with alpha = 0.05. Computations assume that all rows are sample from populations with the same scatter (SD). Number of t tests: 10. Red color indicates if $p < 0.05$. (Figure 4A,B,C).

Concentration (µM)	Multiple t-tests on Detroit 562							
	P value	Mean of selumetinib	Mean of trametinib	Difference	SE of difference	t ratio	df	Adjusted P Value
5.00000	0.009241	66.39	56.79	9.601	3.599	2.668	80.00	0.088660
1.66667	0.002790	74.28	63.17	11.10	3.599	3.086	80.00	0.027554
0.55556	0.000951	77.40	65.05	12.35	3.599	3.432	80.00	0.009474
0.18519	0.000002	83.89	65.44	18.45	3.599	5.126	80.00	0.000020
0.06173	<0.000001	87.16	66.27	20.89	3.599	5.803	80.00	0.000001
0.02058	<0.000001	91.29	70.02	21.28	3.599	5.913	80.00	<0.000001
0.00686	<0.000001	98.70	71.63	27.07	3.599	7.521	80.00	<0.000001
0.00229	<0.000001	100.9	76.45	24.47	3.599	6.800	80.00	<0.000001
0.00076	<0.000001	103.6	75.76	27.87	3.599	7.744	80.00	<0.000001
0.00025	<0.000001	101.9	77.88	24.01	3.599	6.672	80.00	<0.000001

Concentration (µM)	Multiple t-tests on FaDu							
	P value	Mean of selumetinib	Mean of trametinib	Difference	SE of difference	t ratio	df	Adjusted P Value
5.00000	0.029626	63.86	53.85	10.01	4.520	2.215	80.00	0.259729
1.66667	0.001216	70.23	55.06	15.17	4.520	3.355	80.00	0.012092
0.55556	0.000004	79.60	57.28	22.32	4.520	4.938	80.00	0.000042
0.18519	<0.000001	86.26	59.06	27.20	4.520	6.017	80.00	<0.000001
0.06173	<0.000001	90.71	59.12	31.59	4.520	6.987	80.00	<0.000001
0.02058	<0.000001	89.38	61.95	27.43	4.520	6.067	80.00	<0.000001
0.00686	<0.000001	93.15	64.37	28.78	4.520	6.367	80.00	<0.000001
0.00229	0.000004	92.99	70.52	22.47	4.520	4.972	80.00	0.000037
0.00076	0.000010	93.20	71.85	21.36	4.520	4.724	80.00	0.000097
0.00025	<0.000001	101.4	72.96	28.46	4.520	6.295	80.00	<0.000001

Concentration (µM)	Multiple t-tests on SCC25							
	P value	Mean of selumetinib	Mean of trametinib	Difference	SE of difference	t ratio	df	Adjusted P Value
5.00000	0.009852	49.29	32.16	17.14	6.481	2.644	80.00	0.094264

1.66667	0.000002	65.64	32.52	33.12	6.481	5.111	80.00	0.000021
0.55556	<0.000001	71.77	32.66	39.12	6.481	6.036	80.00	<0.000001
0.18519	<0.000001	79.13	34.27	44.86	6.481	6.921	80.00	<0.000001
0.06173	<0.000001	88.18	38.34	49.84	6.481	7.691	80.00	<0.000001
0.02058	<0.000001	98.99	46.24	52.75	6.481	8.140	80.00	<0.000001
0.00686	<0.000001	98.45	51.73	46.72	6.481	7.209	80.00	<0.000001
0.00229	<0.000001	105.9	59.74	46.18	6.481	7.125	80.00	<0.000001
0.00076	<0.000001	98.18	60.74	37.43	6.481	5.776	80.00	0.000001
0.00025	<0.000001	102.7	64.73	37.97	6.481	5.859	80.00	<0.000001

Table S7. Statistical analysis (Multiple t-tests) of effect of trametinib on cell viability. HNSCC cell lines were analyzed in parallel by MTT after 72 h of treatment with trametinib different concentrations. Statistical analysis was performed by Multiple t-tests. The cell viability in all concentration were compared to each other. Statistical significance determined using the Bonferroni-Sidak method, with alpha = 0.05. Computations assume that all rows are sample from populations with the same scatter (SD). Number of t tests: 10. Red color indicates if $p < 0.05$. (Figure 4D).

Concentration (μM)	Multiple t-tests							
	P value	Mean of Detroit 562	Mean of FaDu	Difference	SE of difference	t ratio	df	Adjusted P Value
5.00000	0.451569	56.79	53.85	2.940	3.887	0.7565	80.00	0.997538
1.66667	0.040048	63.17	55.06	8.112	3.887	2.087	80.00	0.335502
0.55556	0.048896	65.05	57.28	7.773	3.887	2.000	80.00	0.394266
0.18519	0.104695	65.44	59.06	6.378	3.887	1.641	80.00	0.669091
0.06173	0.069444	66.27	59.12	7.152	3.887	1.840	80.00	0.513115
0.02058	0.041217	70.02	61.95	8.064	3.887	2.075	80.00	0.343548
0.00686	0.065284	71.63	64.37	7.264	3.887	1.869	80.00	0.490909
0.00229	0.130679	76.45	70.52	5.935	3.887	1.527	80.00	0.753509
0.00076	0.317153	75.76	71.85	3.912	3.887	1.007	80.00	0.977959
0.00025	0.208965	77.88	72.96	4.923	3.887	1.267	80.00	0.904069

Concentration (μM)	Multiple t-tests							
	P value	Mean of Detroit 562	Mean of SCC25	Difference	SE of difference	t ratio	df	Adjusted P Value
5.00000	0.000002	56.79	32.16	24.63	4.825	5.105	80.00	0.000022
1.66667	<0.000001	63.17	32.52	30.66	4.825	6.354	80.00	<0.000001
0.55556	<0.000001	65.05	32.66	32.39	4.825	6.714	80.00	<0.000001
0.18519	<0.000001	65.44	34.27	31.17	4.825	6.460	80.00	<0.000001
0.06173	<0.000001	66.27	38.34	27.94	4.825	5.790	80.00	0.000001
0.02058	0.000004	70.02	46.24	23.78	4.825	4.928	80.00	0.000044
0.00686	0.000090	71.63	51.73	19.91	4.825	4.125	80.00	0.000899
0.00229	0.000860	76.45	59.74	16.71	4.825	3.463	80.00	0.008565
0.00076	0.002576	75.76	60.74	15.02	4.825	3.112	80.00	0.025467
0.00025	0.007877	77.88	64.73	13.15	4.825	2.726	80.00	0.076039

Concentration (μM)	Multiple t-tests							
	P value	Mean of FaDu	Mean of SCC25	Difference	SE of difference	t ratio	df	Adjusted P Value
5.00000	0.000015	53.85	32.16	21.69	4.706	4.609	80.00	0.000151
1.66667	0.000008	55.06	32.52	22.54	4.706	4.790	80.00	0.000075
0.55556	0.000001	57.28	32.66	24.62	4.706	5.231	80.00	0.000013
0.18519	0.000001	59.06	34.27	24.79	4.706	5.268	80.00	0.000011
0.06173	0.000031	59.12	38.34	20.79	4.706	4.416	80.00	0.000311
0.02058	0.001280	61.95	46.24	15.71	4.706	3.339	80.00	0.012724
0.00686	0.008792	64.37	51.73	12.64	4.706	2.686	80.00	0.084526
0.00229	0.024689	70.52	59.74	10.78	4.706	2.289	80.00	0.221194
0.00076	0.020757	71.85	60.74	11.10	4.706	2.359	80.00	0.189219
0.00025	0.084217	72.96	64.73	8.229	4.706	1.748	80.00	0.585116

Table S8. Statistical analysis (Student's t-test) of effects of MEK inhibitors on cell viability. HNSCC cell lines were analyzed in parallel by MTT after 72 h of treatment with MEK inhibitors different concentrations. Statistical analysis was performed by Student's t-test, the IC_{50} concentrations of the MEK inhibitors were compared to each other in each cell line. Red color indicates if $p < 0.05$. (Figure 4E).

Group 1 vs. Group 2	T-test for Independent Samples (MEK_inhibitors_IC50) Note: Variables were treated as independent samples										
	Mean Group 1	Mean Group 2	t-value	df	p	Valid N Group 1	Valid N Group 2	Std.Dev. Group 1	Std.Dev. Group 2	F-ratio Variances	p Variances
Detroit 562 selumetinib IC50 vs. Detroit 562 trametinib IC50	4.789800E+01	3.585000E+20	-1.13855	7	0.292352	5	4	5.815930E+01	7.170000E+20	1.519848E+38	0.000000
FaDu selumetinib IC50 vs. FaDu trametinib IC50	9.697561E+04	3.847400E+01	1.00044	6	0.355723	4	4	1.937896E+05	6.154573E+01	9.914372E+06	0.000000
SCC25 selumetinib IC50 vs. SCC25 trametinib IC50	5.124800E+00	2.755442E-02	2.93095	8	0.018971	5	5	3.888537E+00	4.250748E-02	8.368386E+03	0.000000
Detroit 562 selumetinib IC50 vs. FaDu selumetinib IC50	4.789800E+01	9.697561E+04	-1.13894	7	0.292201	5	4	5.815930E+01	1.937896E+05	1.110255E+07	0.000000
Detroit 562 selumetinib IC50 vs. FaDu trametinib IC50	4.789800E+01	3.847400E+01	0.23558	7	0.820504	5	4	5.815930E+01	6.154573E+01	1.119844E+00	0.880268
Detroit 562 selumetinib IC50 vs. SCC25 selumetinib IC50	4.789800E+01	5.124800E+00	1.64085	8	0.139457	5	5	5.815930E+01	3.888537E+00	2.236999E+02	0.000118
Detroit 562 selumetinib IC50 vs. SCC25 trametinib IC50	4.789800E+01	2.755442E-02	1.84049	8	0.102968	5	5	5.815930E+01	4.250748E-02	1.872007E+06	0.000000
Detroit 562 trametinib IC50 vs. FaDu trametinib IC50	3.585000E+20	3.847400E+01	1.00000	6	0.355918	4	4	7.170000E+20	6.154573E+01	1.357196E+38	0.000000
Detroit 562 trametinib IC50 vs. SCC25 trametinib IC50	3.585000E+20	2.755442E-02	1.13855	7	0.292352	4	5	7.170000E+20	4.250748E-02	2.845166E+44	0.000000
FaDu selumetinib IC50 vs. SCC25 selumetinib IC50	9.697561E+04	5.124800E+00	1.13944	7	0.292005	4	5	1.937896E+05	3.888537E+00	2.483640E+09	0.000000
FaDu selumetinib IC50 vs. SCC25 trametinib IC50	9.697561E+04	2.755442E-02	1.13950	7	0.291982	4	5	1.937896E+05	4.250748E-02	2.078406E+13	0.000000
FaDu trametinib IC50 vs. SCC25 trametinib IC50	3.847400E+01	2.755442E-02	1.42246	7	0.197888	4	5	6.154573E+01	4.250748E-02	2.096356E+06	0.000000

Table S9. Effects of afatinib + trametinib on cell viability – numerical data. HNSCC cell lines were analyzed in parallel by MTT after 72 h of treatment with afatinib + trametinib different concentrations. (Figure 5A,B,C).

Concentration (μM)	Effect of afatinib + trametinib (1:1) on Detroit 562 cell viability (%)						
	Sample 1	Sample 2	Sample 3	Sample 4	Sample 5	Mean	SD
5.00000	41.63369	44.38465	35.29412	44.05766	33.92628	39.85928	4.93192
1.66667	43.54910	44.78505	38.05147	44.95957	29.86012	40.24106	6.45007
0.55556	40.50803	43.25946	37.23262	44.05459	26.23644	38.25823	7.23269
0.18519	38.85320	40.66531	37.50000	44.73153	26.01520	37.55305	7.00057
0.06173	40.02592	41.68320	37.76738	42.69455	27.26413	37.88704	6.22411
0.02058	42.09897	45.19390	40.49131	43.14858	33.72466	40.93148	4.37546
0.00686	45.56888	48.93014	42.69719	46.77261	45.74650	45.94307	2.25417
0.00229	51.21702	54.23420	51.80481	53.11876	54.72987	53.02093	1.51122
0.00076	57.62784	60.88898	53.99398	56.97083	64.15037	58.72640	3.89810
0.00025	62.25853	64.58299	59.55882	63.31390	77.76549	65.49595	7.10399
Concentration (μM)	Effect of afatinib + trametinib (1:1) on FaDu cell viability (%)						
	Sample 1	Sample 2	Sample 3	Sample 4	Sample 5	Mean	SD
5.00000	18.85625	31.76362	35.83758	39.60377	29.75467	31.16318	7.85650
1.66667	32.71249	37.77229	41.33972	45.35491	30.27973	37.49183	6.15568
0.55556	31.36051	37.32741	41.01799	41.04746	31.44503	36.43968	4.84059
0.18519	29.68987	34.56968	38.30949	39.16774	35.39566	35.42649	3.74007
0.06173	28.27896	34.74980	37.55019	35.88989	28.58938	33.01165	4.29705
0.02058	27.64887	35.14792	36.90674	36.75577	33.70681	34.03322	3.80060
0.00686	28.69861	37.28798	39.85182	36.60780	37.99102	36.08745	4.30410
0.00229	31.07407	37.81445	42.37817	36.63059	48.98878	39.37721	6.71410
0.00076	35.31293	44.60271	44.83126	41.94485	63.08003	45.95435	10.31617
0.00025	39.98456	45.56826	53.73979	46.30659	74.79282	52.07840	13.60755
Concentration (μM)	Effect of afatinib + trametinib (1:1) on SCC25 cell viability (%)						
	Sample 1	Sample 2	Sample 3	Sample 4	Sample 5	Mean	SD
5.00000	17.23931	9.23435	7.72845	9.09345	4.32354	9.52382	4.74511
1.66667	23.73868	14.91161	7.03606	11.79496	6.38195	12.77265	7.06283
0.55556	24.08114	18.47751	7.86368	12.86716	7.17951	14.09380	7.19532
0.18519	29.48387	22.66499	10.34654	17.75508	11.95021	18.44014	7.87255
0.06173	34.54787	27.68544	13.58128	22.07189	15.46006	22.66931	8.67733
0.02058	40.36230	30.48883	18.17384	26.66726	19.08134	26.95471	9.10261
0.00686	51.91818	33.36269	19.60185	31.34663	20.50053	31.34598	13.06552
0.00229	64.77843	42.60585	29.04118	35.16234	24.98387	39.31433	15.71141
0.00076	77.90815	46.86379	28.90039	41.86011	28.49226	44.80494	20.17295
0.00025	80.92110	63.27399	32.05938	46.24692	32.03583	50.90744	21.13563

Table S10. Statistical analysis (Multiple t-tests) of effects of afatinib + trametinib on cell viability. HNSCC cell lines were analyzed in parallel by MTT after 72 h of treatment with afatinib + trametinib (1:1) different concentrations. Statistical analysis was performed by Multiple t-tests. The cell viability in all concentration were compared to effects of afatinib, trametinib and afatinib + trametinib in each cell line. Statistical significance determined using the Bonferroni-Sidak method, with alpha = 0.05. Computations assume that all rows are sample from populations with the same scatter (SD). Number of t tests: 10. Red color indicates if $p < 0.05$. (Figure 5A,B,C).

Concentration (μM)	Multiple t-tests on Detroit 562							
	P value	Mean of afatinib	Mean of afatinib + trametinib	Difference	SE of difference	t ratio	df	Adjusted P Value
5.00000	0.168889	32.53	39.86	-7.33	4.846	1.512	80.00	0.136462
1.66667	0.042718	51.63	40.24	11.39	4.731	2.407	80.00	0.038351
0.55556	0.011602	53.89	38.26	15.63	4.801	3.256	80.00	0.018748
0.18519	0.005867	54.81	37.55	17.25	4.637	3.721	80.00	0.011851
0.06173	0.003605	56.44	37.89	18.56	4.565	4.066	80.00	0.00971
0.02058	0.000153	59.99	40.93	19.06	2.846	6.696	80.00	0.001239
0.00686	0.0008	63.09	45.94	17.14	3.283	5.223	80.00	0.003233

0.00229	0.020534	72.07	53.02	19.05	6.617	2.879	80.00	0.027652
0.00076	0.026806	75.19	58.73	16.46	6.083	2.706	80.00	0.030942
0.00025	0.042483	81.84	65.5	16.34	6.779	2.41	80.00	0.038351

Multiple t-tests on Detroit 562

Concentration (μM)	P value	Mean of trametinib	Mean of afatinib + trametinib	Difference	SE of difference	t ratio	df	Adjusted P Value
5.00000	0.000292	56.79	39.86	-16.93	2.779	6.092	80.00	0.000042
1.66667	0.005161	63.17	40.24	-22.93	6.019	3.81	80.00	0.000579
0.55556	0.000372	65.05	38.26	-26.79	4.561	5.874	80.00	0.000047
0.18519	0.000168	65.44	37.55	-27.89	4.222	6.606	80.00	0.000028
0.06173	0.000113	66.27	37.89	-28.39	4.056	6.999	80.00	0.000023
0.02058	0.000006	70.02	40.93	-29.08	2.763	10.53	80.00	0.000003
0.00686	0.000014	71.63	45.94	-25.69	2.76	9.307	80.00	0.000005
0.00229	<0,000001	76.45	53.02	-23.43	1.417	16.53	80.00	<0,000001
0.00076	0.000105	75.76	58.73	-17.03	2.409	7.069	80.00	0.000023
0.00025	0.021044	77.88	65.5	-12.39	4.325	2.863	80.00	0.002125

Multiple t-tests on FaDu

Concentration (μM)	P value	Mean of afatinib	Mean of afatinib + trametinib	Difference	SE of difference	t ratio	df	Adjusted P Value
5.00000	0.000092	5.458	31.16	-25.7	3.566	7.208	80.00	0.000112
1.66667	0.216819	42.01	37.49	4.522	3.373	1.341	80.00	0.065696
0.55556	0.00052	51.73	36.44	15.29	2.738	5.584	80.00	0.000315
0.18519	0.000083	53.05	35.43	17.63	2.41	7.315	80.00	0.000112
0.06173	0.000308	50.34	33.01	17.33	2.867	6.044	80.00	0.000233
0.02058	0.000111	51.54	34.03	17.51	2.497	7.014	80.00	0.000112
0.00686	0.000765	52.21	36.09	16.12	3.065	5.26	80.00	0.000386
0.00229	0.003867	55.67	39.38	16.29	4.058	4.015	80.00	0.001674
0.00076	0.024119	61.22	45.95	15.27	5.502	2.775	80.00	0.009135
0.00025	0.039898	70.31	52.08	18.23	7.44	2.451	80.00	0.013432

Multiple t-tests on FaDu

Concentration (μM)	P value	Mean of trametinib	Mean of afatinib + trametinib	Difference	SE of difference	t ratio	df	Adjusted P Value
5.00000	0.000842	53.85	31.16	-22.69	4.379	5.181	80.00	0.000106
1.66667	0.000261	55.06	37.49	-17.57	2.836	6.196	80.00	0.000038
0.55556	0.000149	57.28	36.44	-20.84	3.1	6.722	80.00	0.000025
0.18519	0.000136	59.06	35.43	-23.64	3.468	6.815	80.00	0.000025
0.06173	0.000023	59.12	33.01	-26.11	2.982	8.757	80.00	0.000014
0.02058	0.000104	61.95	34.03	-27.92	3.943	7.081	80.00	0.000025
0.00686	0.000028	64.37	36.09	-28.28	3.325	8.506	80.00	0.000014
0.00229	0.000149	70.52	39.38	-31.14	4.632	6.723	80.00	0.000025
0.00076	0.000991	71.85	45.95	-25.89	5.128	5.049	80.00	0.000111
0.00025	0.012774	72.96	52.08	-20.88	6.542	3.192	80.00	0.00129

Multiple t-tests on SCC25

Concentration (μM)	P value	Mean of afatinib	Mean of afatinib + trametinib	Difference	SE of difference	t ratio	df	Adjusted P Value
5.00000	0.002693	24.59	9.524	15.07	3.522	4.278	80.00	0.006347
1.66667	0.000369	40.57	12.77	27.8	4.725	5.882	80.00	0.002609
0.55556	0.000845	41.46	14.09	27.36	5.284	5.178	80.00	0.002986
0.18519	0.004594	39.5	18.44	21.06	5.41	3.892	80.00	0.008119
0.06173	0.020923	40.45	22.67	17.78	6.203	2.867	80.00	0.029585
0.02058	0.056282	40.83	26.95	13.87	6.22	2.23	80.00	0.066319
0.00686	0.129548	43.93	31.35	12.59	7.45	1.69	80.00	0.114488
0.00229	0.191925	52.02	39.31	12.71	8.915	1.425	80.00	0.146732
0.00076	0.207542	59.32	44.8	14.52	10.59	1.371	80.00	0.146732
0.00025	0.095405	71.46	50.91	20.55	10.87	1.89	80.00	0.09636

Multiple t-tests on SCC25

Concentration (μM)	P value	Mean of trametinib	Mean of afatinib + trametinib	Difference	SE of difference	t ratio	df	Adjusted P Value
5.00000	0.000169	32.16	9.524	-22.63	3.428	6.602	80.00	0.001195
1.66667	0.002259	32.52	12.77	-19.74	4.478	4.409	80.00	0.005324
0.55556	0.00188	32.66	14.09	-18.56	4.082	4.548	80.00	0.005324
0.18519	0.009454	34.27	18.44	-15.83	4.665	3.393	80.00	0.011548
0.06173	0.009801	38.34	22.67	-15.67	4.651	3.369	80.00	0.011548
0.02058	0.006469	46.24	26.95	-19.28	5.279	3.653	80.00	0.011434
0.00686	0.02326	51.73	31.35	-20.38	7.283	2.798	80.00	0.023493
0.00229	0.049277	59.74	39.31	-20.43	8.823	2.315	80.00	0.043548
0.00076	0.153803	60.74	44.8	-15.94	10.12	1.575	80.00	0.120821
0.00025	0.245692	64.73	50.91	-13.82	11.03	1.253	80.00	0.173704

Table S11. Statistical analysis (Student's t-test) of effects of afatinib + trametinib on cell viability. HNSCC cell lines were analyzed in parallel by MTT after 72 h of treatment with afatinib + trametinib (1:1) different concentrations. Statistical analysis was performed by Student's t-test, the IC_{50} concentrations of the afatinib, trametinib and afatinib + trametinib were compared to each other in each cell line. Red color indicates if $p < 0.05$. (Figure 5D).

Group 1 vs. Group 2	T-test for Independent Samples (Combination_IC50) Note: Variables were treated as independent samples										
	Mean Group 1	Mean Group 2	t-value	df	p	Valid N Group 1	Valid N Group 2	Std.Dev. Group 1	Std.Dev. Group 2	F-ratio Variances	p Variances
Detroit 562 afatinib IC50 vs. Detroit 562 afatinib + trametinib IC50	4.255650E-01	9.379600E-03	2.78902	7	0.026946	4	5	3.396928E-01	7.231761E-03	2.206401E+03	0.000001
Detroit 562 trametinib IC50 vs. Detroit 562 afatinib + trametinib IC50	3.585000E+20	9.379600E-03	1.13855	7	0.292352	4	5	7.170000E+20	7.231761E-03	9.829923E+45	3.931969E+45
FaDu afatinib IC50 vs. FaDu afatinib + trametinib IC50	4.275600E-02	1.019429E-03	2.85760	7	0.024421	5	4	2.874869E-02	2.029723E-03	2.006145E+02	0.001134
FaDu trametinib IC50 vs. FaDu afatinib + trametinib IC50	3.847400E+01	1.019429E-03	1.25022	6	0.257767	4	4	6.154573E+01	2.029723E-03	9.194373E+08	0.000000
SCC25 afatinib IC50 vs. SCC25 afatinib + trametinib IC50	5.834378E-02	2.923341E-03	1.05980	8	0.320180	5	5	1.167777E-01	5.983802E-03	3.808604E+02	0.000041
SCC25 trametinib IC50 vs. SCC25 afatinib + trametinib IC50	2.755442E-02	2.923341E-03	1.28305	8	0.235396	5	5	4.250748E-02	5.983802E-03	5.046338E+01	0.002236

Table S12. Expression data of pY1068-EGFR, EGFR, pS473-Akt, Akt, pT202/Y204-ERK and ERK in HNSCC cell lines. Densitometry analysis was performed using three independent experiments. The expression of all proteins were normalized to the expression of α-tubulin. (Figure 6B).

Cell line	Densitometry intensity of α-tubulin				
	Sample 1	Sample 2	Sample 3	Mean	SD
Detroit 562	26528	22227	30829	26528	4301
FaDu	31305	28803	33806	31305	2501
SCC25	18995	15800	22191	18995	3196
**Cell line**	**Densitometry intensity of pY1068-EGFR**				
	Sample 1	Sample 2	Sample 3	Mean	SD
Detroit 562	13541	13567	18997	15368	3143
FaDu	5640	7110	10109	7620	2278
SCC25	10069	13641	15472	13061	2748
**Cell line**	**Densitometry intensity ratio of pY1068-EGFR**				
	Sample 1	Sample 2	Sample 3	Mean	SD
Detroit 562	0.51	0.61	0.62	0.58	0.06
FaDu	0.18	0.25	0.30	0.24	0.06
SCC25	0.53	0.86	0.70	0.70	0.17
**Cell line**	**Densitometry intensity of EGFR**				
	Sample 1	Sample 2	Sample 3	Mean	SD
Detroit 562	32921	19913	31474	28103	7129
FaDu	28413	18679	30011	25701	6134
SCC25	27145	14954	31723	24607	8668
**Cell line**	**Densitometry intensity ratio of EGFR**				
	Sample 1	Sample 2	Sample 3	Mean	SD
Detroit 562	1.24	0.90	1.02	1.05	0.17
FaDu	0.91	0.65	0.89	0.81	0.14
SCC25	1.43	0.95	1.43	1.27	0.28
**Cell line**	**Densitometry intensity of pS473-Akt**				
	Sample 1	Sample 2	Sample 3	Mean	SD
Detroit 562	21057	15893	23630	20193	3940
FaDu	18099	17558	22363	19340	2632
SCC25	14905	8464	15857	13075	4022
**Cell line**	**Densitometry intensity ratio of pS473-Akt**				
	Sample 1	Sample 2	Sample 3	Mean	SD
Detroit 562	0.79	0.72	0.77	0.76	0.04
FaDu	0.58	0.61	0.66	0.62	0.04
SCC25	0.78	0.54	0.71	0.68	0.13
**Cell line**	**Densitometry intensity of Akt**				
	Sample 1	Sample 2	Sample 3	Mean	SD
Detroit 562	15705	11170	10911	12595	2696
FaDu	19102	25444	16896	20480	4437
SCC25	17353	6984	16907	13748	5862
**Cell line**	**Densitometry intensity ratio of Akt**				
	Sample 1	Sample 2	Sample 3	Mean	SD
Detroit 562	0.59	0.50	0.35	0.48	0.12
FaDu	0.61	0.88	0.50	0.66	0.20
SCC25	0.91	0.44	0.76	0.71	0.24
**Cell line**	**Densitometry intensity of pT202/Y204-ERK**				
	Sample 1	Sample 2	Sample 3	Mean	SD
Detroit 562	14918	3169	10934	9674	5975
FaDu	25678	17140	11963	18260	6926
SCC25	20803	15749	15416	17323	3019

Cell line	Densitometry intensity ratio of pT202/Y204-ERK				
	Sample 1	Sample 2	Sample 3	Mean	SD
Detroit 562	0.56	0.14	0.35	0.35	0.21
FaDu	0.82	0.60	0.35	0.59	0.23
SCC25	1.10	1.00	0.69	0.93	0.21
Cell line	Densitometry intensity of ERK				
	Sample 1	Sample 2	Sample 3	Mean	SD
Detroit 562	22634	21839	23786	22753	979
FaDu	27385	28855	22540	26260	3305
SCC25	15346	18662	18746	17585	1939
Cell line	Densitometry intensity ratio of ERK				
	Sample 1	Sample 2	Sample 3	Mean	SD
Detroit 562	0.85	0.98	0.77	0.87	0.11
FaDu	0.87	1.00	0.67	0.85	0.17
SCC25	0.81	1.18	0.84	0.94	0.21

Table S13. Statistical analysis of expression of pY1068-EGFR, EGFR, pS473-Akt, Akt, pT202/Y204-ERK and ERK in HNSCC cell lines. Statistical analysis was performed by Student's t-test, the pY1068-EGFR, EGFR, pS473-Akt, Akt, pT202/Y204-ERK and ERK expression of the cell lines were compared to each other. Red color indicates if $p < 0.05$. (Figure 6B).

Group 1 vs. Group 2	T-test for Independent Samples (Protein_expression) Note: Variables were treated as independent samples										
	Mean Group 1	Mean Group 2	t-value	df	p	Valid N Group 1	Valid N Group 2	Std.Dev. Group 1	Std.Dev. Group 2	F-ratio Variances	p Variances
Detroit 562 pY1068-EGFR vs. FaDu pY1068-EGFR	0.579008	0.242016	6.9346	4	0.002271	3	3	0.059450	0.059584	1.00452	0.997745
Detroit 562 pY1068-EGFR vs. SCC25 pY1068-EGFR	0.579008	0.696898	-1.1541	4	0.312726	3	3	0.059450	0.166643	7.85730	0.225802
FaDu pY1068-EGFR vs. SCC25 pY1068-EGFR	0.242016	0.696898	-4.4519	4	0.011230	3	3	0.059584	0.166643	7.82195	0.226707
Detroit 562 EGFR vs. FaDu EGFR	1.052619	0.814624	1.8196	4	0.142937	3	3	0.174711	0.144207	1.46780	0.810439
Detroit 562 EGFR vs. SCC25 EGFR	1.052619	1.268339	-1.1357	4	0.319508	3	3	0.174711	0.278766	2.54589	0.564034
FaDu EGFR vs. SCC25 EGFR	0.814624	1.268339	-2.5039	4	0.066491	3	3	0.144207	0.278766	3.73685	0.422222
Detroit 562 pS473-Akt vs. FaDu pS473-Akt	0.758424	0.616412	4.2375	4	0.013290	3	3	0.039975	0.042089	1.10859	0.948502
Detroit 562 pS473-Akt vs. SCC25 pS473-Akt	0.758424	0.678316	1.0320	4	0.360386	3	3	0.039975	0.128370	10.31235	0.176798
FaDu pS473-Akt vs. SCC25 pS473-Akt	0.616412	0.678316	-0.7937	4	0.471806	3	3	0.042089	0.128370	9.30224	0.194133
Detroit 562 Akt vs. FaDu Akt	0.482831	0.664442	-1.3606	4	0.245266	3	3	0.120262	0.197456	2.69579	0.541156
Detroit Akt vs. SCC25 Akt	0.482831	0.705821	-1.4354	4	0.224502	3	3	0.120262	0.240702	4.00596	0.399524
FaDu Akt vs. SCC25 Akt	0.664442	0.705821	-0.2302	4	0.829226	3	3	0.197456	0.240702	1.48600	0.804504
Detroit 562 pT202/Y204-ERK vs. FaDu pT202/Y204-ERK	0.353208	0.589726	-1.3056	4	0.261717	3	3	0.209888	0.233238	1.23488	0.894902
Detroit 562 pT202/Y204-ERK vs. SCC25 pT202/Y204-ERK	0.353208	0.928898	-3.3688	4	0.028075	3	3	0.209888	0.208703	1.01138	0.994340
FaDu pT202/Y204-ERK vs. SCC25 pT202/Y204-ERK	0.589726	0.928898	-1.8770	4	0.133745	3	3	0.233238	0.208703	1.24894	0.889308
Detroit 562 ERK vs. FaDu ERK	0.869107	0.847775	0.1849	4	0.862313	3	3	0.106414	0.169158	2.52689	0.567072
Detroit 562 ERK vs. SCC25 ERK	0.869107	0.944607	-0.5647	4	0.602470	3	3	0.106414	0.205697	3.73644	0.422258
FaDu ERK vs. SCC25 ERK	0.847775	0.944607	-0.6298	4	0.563028	3	3	0.169158	0.205697	1.47867	0.806885

Table S14. Expression data of pY1068-EGFR, EGFR, pS473-Akt, Akt, pT202/Y204-ERK and ERK after trametinib treatment in HNSCC cell lines. Densitometry analysis was performed using three independent experiments. The expression of all proteins were compared to expression of DMSO treated negative controls, after normalization to α-tubulin. (Figure 7B).

Cell line	Treatment		Densitometry intensity of α-tubulin				
	DMSO	trametinib	Sample 1	Sample 2	Sample 3	Mean	SD
Detroit 562	+	-	11288	13487	12982	12585	1152
	-	+	14352	14297	10090	12913	2445
FaDu	+	-	19828	15308	20539	18558	2837
	-	+	23361	16435	11154	16983	6122
SCC25	+	-	14587	14587	14846	14674	150
	-	+	13641	13641	13171	13485	272
Cell line	Treatment		Densitometry intensity of pY1068-EGFR				
	DMSO	trametinib	Sample 1	Sample 2	Sample 3	Mean	SD
Detroit 562	+	-	18876	18338	18338	18517	311
	-	+	19579	20642	18642	19621	1001
FaDu	+	-	10475	9152	11103	10243	996
	-	+	24316	18820	18699	20612	3209
SCC25	+	-	23246	35036	7805	22029	13656
	-	+	24608	30297	8209	21038	11469
Cell line	Treatment		Densitometry intensity ratio of pY1068-EGFR				
	DMSO	trametinib	Sample 1	Sample 2	Sample 3	Mean	SD
Detroit 562	+	-	1.00	1.00	1.00	1.00	0.00
	-	+	0.82	1.06	1.31	1.06	0.25
FaDu	+	-	1.00	1.00	1.00	1.00	0.00
	-	+	1.97	1.92	3.10	2.33	0.67
SCC25	+	-	1.00	1.00	1.00	1.00	0.00
	-	+	1.13	0.92	1.19	1.08	0.14
Cell line	Treatment		Densitometry intensity of EGFR				
	DMSO	trametinib	Sample 1	Sample 2	Sample 3	Mean	SD
Detroit 562	+	-	20946	28149	25343	24813	3630
	-	+	24250	22509	24181	23647	986
FaDu	+	-	29233	23864	22749	25282	3467
	-	+	28797	25359	16183	23446	6521
SCC25	+	-	34561	33916	19550	29342	8487
	-	+	33939	27623	17799	26454	8133
Cell line	Treatment		Densitometry intensity ratio of EGFR				
	DMSO	trametinib	Sample 1	Sample 2	Sample 3	Mean	SD
Detroit 562	+	-	1.00	1.00	1.00	1.00	0.00
	-	+	0.91	0.75	1.23	0.96	0.24
FaDu	+	-	1.00	1.00	1.00	1.00	0.00
	-	+	0.84	0.99	1.31	1.05	0.24
SCC25	+	-	1.00	1.00	1.00	1.00	0.00
	-	+	1.05	0.87	1.03	0.98	0.10
Cell line	Treatment		Densitometry intensity of pS473-Akt				
	DMSO	trametinib	Sample 1	Sample 2	Sample 3	Mean	SD
Detroit 562	+	-	26482	29217	29217	28305	1579
	-	+	31673	24543	24543	26920	4116
FaDu	+	-	11385	7470	10220	9692	2010
	-	+	26992	18242	14350	19861	6475
SCC25	+	-	12339	12339	32144	18941	11434
	-	+	12775	12775	32326	19292	11288
Cell line	Treatment		Densitometry intensity ratio of pS473-Akt				
	DMSO	trametinib	Sample 1	Sample 2	Sample 3	Mean	SD
Detroit 562	+	-	1.00	1.00	1.00	1.00	0.00

Cell line	DMSO	trametinib	Sample 1	Sample 2	Sample 3	Mean	SD
	-	+	0.94	0.79	1.08	0.94	0.14
FaDu	+	-	1.00	1.00	1.00	1.00	0.00
	-	+	2.01	2.27	2.59	2.29	0.29
SCC25	+	-	1.00	1.00	1.00	1.00	0.00
	-	+	1.11	1.11	1.13	1.12	0.02

Cell line	Treatment		Densitometry intensity of Akt				
	DMSO	trametinib	Sample 1	Sample 2	Sample 3	Mean	SD
Detroit 562	+	-	8937	17015	13908	13287	4075
	-	+	12662	17223	9068	12984	4087
FaDu	+	-	25174	19665	25128	23323	3168
	-	+	21964	21596	20769	21443	612
SCC25	+	-	18339	18339	4636	13771	7911
	-	+	18068	18068	5331	13822	7353

Cell line	Treatment		Densitometry intensity ratio of Akt				
	DMSO	trametinib	Sample 1	Sample 2	Sample 3	Mean	SD
Detroit 562	+	-	1.00	1.00	1.00	1.00	0.00
	-	+	1.11	0.95	0.84	0.97	0.14
FaDu	+	-	1.00	1.00	1.00	1.00	0.00
	-	+	0.74	1.02	1.52	0.10	0.40
SCC25	+	-	1.00	1.00	1.00	1.00	0.00
	-	+	1.05	1.05	1.30	1.13	0.14

Cell line	Treatment		Densitometry intensity of pT202/Y204-ERK				
	DMSO	trametinib	Sample 1	Sample 2	Sample 3	Mean	SD
Detroit 562	+	-	18050	15823	42804	25559	14976
	-	+	267	260	227	251	22
FaDu	+	-	6118	17789	40102	21336	17267
	-	+	258	248	366	291	65
SCC25	+	-	8418	15370	12931	12240	3527
	-	+	176	216	231	207	29

Cell line	Treatment		Densitometry intensity ratio of pT202/Y204-ERK				
	DMSO	trametinib	Sample 1	Sample 2	Sample 3	Mean	SD
Detroit 562	+	-	1.00	1.00	1.00	1.00	0.00
	-	+	0.01	0.02	0.01	0.01	0.00
FaDu	+	-	1.00	1.00	1.00	1.00	0.00
	-	+	0.04	0.01	0.02	0.02	0.01
SCC25	+	-	1.00	1.00	1.00	1.00	0.00
	-	+	0.02	0.02	0.02	0.02	0.00

Cell line	Treatment		Densitometry intensity of ERK				
	DMSO	trametinib	Sample 1	Sample 2	Sample 3	Mean	SD
Detroit 562	+	-	37254	44774	41720	41249	3782
	-	+	43701	41497	35087	40095	4475
FaDu	+	-	21374	20216	26537	22709	3365
	-	+	27053	23029	19860	23314	3605
SCC25	+	-	39034	35589	39723	38115	2215
	-	+	34698	37786	39893	37459	2613

Cell line	Treatment		Densitometry intensity ratio of ERK				
	DMSO	trametinib	Sample 1	Sample 2	Sample 3	Mean	SD
Detroit 562	+	-	1.00	1.00	1.00	1.00	0.00
	-	+	0.92	0.87	1.08	0.96	0.11
FaDu	+	-	1.00	1.00	1.00	1.00	0.00
	-	+	1.07	1.06	1.38	1.17	0.18
SCC25	+	-	1.00	1.00	1.00	1.00	0.00
	-	+	0.95	1.14	1.13	1.07	0.11

Table S15. Statistical analysis of expression of pY1068-EGFR, EGFR, pS473-Akt, Akt, pT202/Y204-ERK and ERK after trametinib treatment in HNSCC cell lines. Statistical analysis was performed by Student's t-test, in each cell line the expression of all proteins in trametinib treated samples were compared to protein expression in DMSO treated samples. Red color indicates if $p < 0.05$. (Figure 7B).

Group 2 vs. Group 1	T-test for Independent Samples (Treatment) Note: Variables were treated as independent samples										
	Mean Group 1	Mean Group 2	t-value	df	p	Valid N Group 1	Valid N Group 2	Std.Dev. Group 1	Std.Dev. Group 2	F-ratio Variances	p Variances
Detroit 562: DMSO pY1068-EGFR vs. trametinib pY1068-EGFR	1.061832	1.000000	0.435	4	0.686050	3	3	0.246036	0.010000	605.338	0.003298
FaDu: DMSO pY1068-EGFR vs. trametinib pY1068-EGFR	2.328908	1.000000	3.438	4	0.026344	3	3	0.669443	0.010000	4481.541	0.000446
SCC25: DMSO pY1068-EGFR vs. trametinib pY1068-EGFR	1.080725	1.000000	1.012	4	0.368585	3	3	0.137736	0.010000	189.713	0.010487
Detroit 562: DMSO EGFR vs. trametinib EGFR	0.964149	1.000000	-0.257	4	0.809655	3	3	0.241143	0.010000	581.501	0.003433
FaDu: DMSO EGFR vs. trametinib EGFR	1.045258	1.000000	0.324	4	0.762177	3	3	0.241738	0.010000	584.375	0.003417
SCC25: DMSO EGFR vs. trametinib EGFR	0.982409	1.000000	-0.312	4	0.770968	3	3	0.097295	0.010000	94.664	0.020906
Detroit 562: DMSO pS473-Akt vs. trametinib pS473-Akt	0.937925	1.000000	-0.744	4	0.498286	3	3	0.144195	0.010000	207.922	0.009573
FaDu: DMSO pS473-Akt vs. trametinib pS473-Akt	2.290709	1.000000	7.788	4	0.001466	3	3	0.286889	0.010000	823.054	0.002427
SCC25: DMSO pS473-Akt vs. trametinib pS473-Akt	1.115925	1.000000	10.989	4	0.000390	3	3	0.015292	0.010000	2.338	0.599087
Detroit 562: DMSO Akt vs. trametinib Akt	0.969316	1.000000	-0.383	4	0.721073	3	3	0.138331	0.010000	191.356	0.010397
FaDu: DMSO Akt vs. trametinib Akt	1.095125	1.000000	0.416	4	0.698592	3	3	0.395697	0.010000	1565.758	0.001277
SCC25: DMSO Akt vs. trametinib Akt	1.134395	1.000000	1.658	4	0.172729	3	3	0.140071	0.010000	196.198	0.010142
Detroit 562: DMSO pT202/Y204-ERK vs. trametinib pT202/Y204-ERK	0.011324	1.000000	-156.949	4	0.000000	3	3	0.004364	0.010000	5.251	0.319972
FaDu: DMSO pT202/Y204-ERK vs. trametinib pT202/Y204-ERK	0.021880	1.000000	-107.111	4	0.000000	3	3	0.012254	0.010000	1.502	0.799448
SCC25: DMSO pT202/Y204-ERK vs. trametinib pT202/Y204-ERK	0.019147	1.000000	-159.184	4	0.000000	3	3	0.003728	0.010000	7.193	0.244097
Detroit 562: DMSO ERK vs. trametinib ERK	0.959624	1.000000	-0.641	4	0.556691	3	3	0.108725	0.010000	118.211	0.016777
FaDu: DMSO ERK vs. trametinib ERK	1.171131	1.000000	1.650	4	0.174295	3	3	0.179367	0.010000	321.724	0.006197
SCC25: DMSO ERK vs. trametinib ERK	1.072646	1.000000	1.185	4	0.301711	3	3	0.105736	0.010000	111.802	0.017730

Table S16. Data tables summarizing of EGFR and pY1068-EGFR immunohistochemical analysis.

	EGFR expression		
Primary tumor site	low	high	Total
Oropharynx	1	32	33
Hypopharynx	2	28	30
Supraglottis	3	16	19
Glottis	0	2	2
Total	6	78	84

	pY1068-EGFR expression		
Primary tumor site	low	high	Total
Oropharynx	19	3	22
Hypopharynx	21	1	22
Supraglottis	16	2	18
Glottis	1	0	1
Total	57	6	63

	EGFR expression		
TNM[1] T parameter	low	high	Total
1	0	13	13
2	2	24	26
3	2	21	23
4a	2	14	16
4b	0	6	6
Total	6	78	84

	pY1068-EGFR expression		
TNM[1] T parameter	low	high	Total
1	9	0	9
2	17	2	19
3	21	1	22
4a	8	2	10
4b	2	1	3
Total	57	6	63

	EGFR expression		
TNM[1] N parameter	low	high	Total
0	2	36	38
1	0	15	15
2a	1	5	6
2b	1	9	10
2c	2	10	12
3	0	3	3
Total	6	78	84

	pY1068-EGFR expression		
TNM[1] N parameter	low	high	Total
0	26	1	27
1	11	1	12
2a	4	0	4
2b	8	1	9
2c	7	1	8
3	1	2	3
Total	57	6	63

	EGFR expression		
TNM[1] M parameter	low	high	Total
0	6	0	6
1	73	5	78
Total	79	5	84

TNM[1] M parameter	pY1068-EGFR expression low	high	Total
0	53	4	57
1	5	1	6
Total	58	5	63

TNM[1] stage	EGFR expression low	high	Total
1	0	24	24
2	6	49	55
3	0	5	5
Total	6	78	84

TNM[1] stage	pY1068-EGFR expression low	high	Total
1	16	0	16
2	37	5	42
3	4	1	5
Total	57	6	63

Grade	EGFR expression low	high	Total
1	1	4	5
2	0	38	38
3	5	26	31
Total	6	68	74

Grade	pY1068-EGFR expression low	high	Total
1	5	0	5
2	25	3	28
3	20	2	22
Total	50	5	55

Tobacco use	EGFR expression low	high	Total
never	1	8	9
previously yes	2	23	25
currently	3	45	48
Total	6	76	82

Tobacco use	pY1068-EGFR expression low	high	Total
never	6	0	6
previously yes	15	0	15
currently	35	6	41
Total	56	6	62

Alcohol use	EGFR expression low	high	Total
never	1	19	20
previously yes	3	26	29
currently	2	33	35
Total	6	78	84

Alcohol use	pY1068-EGFR expression low	high	Total
never	12	1	13
previously yes	20	2	22
currently	25	3	28
Total	57	6	63

[1] TNM: tumor, node and metastasis, UICC TNM 7th edition.
